# 1,4-Dihydropyridine Derivatives: Dihydronicotinamide Analogues—Model Compounds Targeting Oxidative Stress

**DOI:** 10.1155/2016/1892412

**Published:** 2016-01-06

**Authors:** Astrida Velena, Neven Zarkovic, Koraljka Gall Troselj, Egils Bisenieks, Aivars Krauze, Janis Poikans, Gunars Duburs

**Affiliations:** ^1^Laboratory of Membrane Active Compounds and Beta-Diketones, Latvian Institute of Organic Synthesis, Riga LV-1006, Latvia; ^2^Ruđer Bošković Institute, Bijenička cesta 54, 10000 Zagreb, Croatia

## Abstract

Many 1,4-dihydropyridines (DHPs) possess redox properties. In this review DHPs are surveyed as protectors against oxidative stress (OS) and related disorders, considering the DHPs as specific group of potential antioxidants with bioprotective capacities. They have several peculiarities related to antioxidant activity (AOA). Several commercially available calcium antagonist, 1,4-DHP drugs, their metabolites, and calcium agonists were shown to express AOA. Synthesis, hydrogen donor properties, AOA, and methods and approaches used to reveal biological activities of various groups of 1,4-DHPs are presented. Examples of DHPs antioxidant activities and protective effects of DHPs against OS induced damage in low density lipoproteins (LDL), mitochondria, microsomes, isolated cells, and cell cultures are highlighted. Comparison of the AOA of different DHPs and other antioxidants is also given. According to the data presented, the DHPs might be considered as bellwether among synthetic compounds targeting OS and potential pharmacological model compounds targeting oxidative stress important for medicinal chemistry.

## 1. Introduction

1,4-Dihydropyridines (DHPs) [1], including Ca^2+^ antagonist (CA) drugs [2], are large group of structurally diverse compounds. Functionally, they are similar to dihydronicotinamide redox-active synthetic compounds with radical scavenging and antioxidant (AO) properties and may be considered as protectors against oxidative stress (OS) and associated disorders [3].

Oxidative stress is extremely important for molecular pathogenesis, especially influencing the redox regulation of cellular signaling pathways [4–7]. Oxidative stress closely relates to presence of oxygen and nitrogen free radicals, known as reactive oxygen species and reactive nitrogen species (ROS and RNS, resp.). They cumulatively increase upon cellular exposure to various endogenous and/or exogenous insults. ROS and RNS have the “two-faced” character and play a dual role as both deleterious and beneficial species [8, 9]. Although explored in many diseases, various phenomena related to OS have been probably best studied in cancer cells in which, depending on various factors, OS may have anticancer-like effects. Its protumorigenic effects are primarily related to induction of oxidative DNA lesions (8-OH-G) and consequential increase of DNA mutations that may, if not repaired, lead to genome instability and an increased rate of cellular proliferation [10]. On the other hand, antitumorigenic actions of OS have been closely linked to cellular processes of senescence and apoptosis, two major molecular mechanisms that counteract tumor development. Which of these two actions will dominate depends on many factors including the metabolic status of the cell, as recently reviewed by Kujundžić et al., 2014 [11].

Antioxidants (AOs) are defined as substances that, even when present in low concentrations compared to those of an oxidizable substrate, prevent or significantly delay the oxidation process (Halliwell and Gutteridge, 1995 [12]). Their activity depends on complex factors including the nature of the antioxidants, the condition of oxidation, the properties of substrate oxidized, and the level of oxidation (reviewed in Kancheva and Kasaikina, 2013 [13]). Accordingly, an antioxidative effect may be direct, resulting from direct ROS scavenging, or indirect from the influence on various signaling pathways related to cellular defense, that is, stress responses. In relation to human physiology, antioxidants are traditionally classified as exogenous (supplied mostly through food) and endogenous and are further subclassified as enzymatic (i.e., superoxide dismutase (SOD) and catalase (CAT)) and nonenzymatic (i.e., glutathione, vitamins A, C, and E, etc.) [3].

DHPs could be classified as the separate group of synthetic nonenzymatic, however, biomimetic AOs.

## 2. Oxidative Stress and Its Prevention: Wavy Scientific Process Development—*Pro et Contra*

There are opposite views both towards the role of oxidative stress and about potential applications of exogenous antioxidants in onset of OS [14–16].

Herewith, we need to mention that antioxidants have been studied for decades (starting from 1970s) as the tools for the treatment of various disorders. The role of native and synthetic antioxidants (acting on lipid peroxidation (LP) in biological membranes) in radiation damage and malignant growth was seriously evaluated [17]. The overall conclusions point out antioxidants role in decreasing the damage of cells by reducing oxidants before the occurrence of cellular damage [14]. It was elicited and accented (Burlakova et al. [15]) that

- antioxidants, nontoxic inhibitors of free radical processes, exhibit a wide gamut (pleiotropy) of biological activity (as further will be reported, this phenomenon is also characteristic for the DHP antioxidants group);
- the biological effectiveness of AOs correlates with their antioxidant activity (AOA);
- depending on dose, AOs may either increase or decrease the AOA;
- the efficacy of AO depends on the time of introduction in the course of medical treatment because the development of the disease may be accompanied by stages of changing the AOA.

In relation to dose-effect dependence, Burlakova et al. [15] have found the nonlinear pattern: after addition of an AO, there is an initial increase of AOA, followed by returning to normal and finally decreasing drastically below the normal value. Therefore, antioxidants may produce a specific effect by decreasing (at low doses) or increasing (at high doses) the rate of free radical reactions. Hence, the compound may be efficient AO only if it is introduced in a low dose at the stage of reduced AOA or in a high dose at the stage of AOA elevation. The widespread opinion of opponents was that the antioxidant function, even that of tocopherol, was a side effect of its activity and important only for* in vitro* processes and without any role in bioobjects life. This opinion was supported by the fact that the deficiency of natural AO tocopherol (E-avitaminosis) cannot be cured completely by applying synthetic AO. Eventually, it was not certain also that detected lipid peroxides have been generated* in vivo* in the intact organs and were not artificially formed during the isolation [15]. All these objections and skepticism were rejected in due time.

However, some other research directions were suggested.

Fang et al. [18] reported two different therapeutic strategies for modulating OS in cancer and inflammation, including (1) antioxidant therapy and (2) “oxidation therapy.”

For (1), polymeric superoxide dismutase (e.g., pyran copolymer-SOD), xanthine oxidase (XO) inhibitor, developed water-soluble form of 4-amino-6-hydroxypyrazolo[3,4-*d*]pyrimidine (AHPP), heme oxygenase-1 (HO-1) inducers (e.g., hemin and its polymeric form), and other antioxidants or radical scavengers (e.g., phenolic compound canolol, 4-vinyl-2,6-dimethoxyphenol) were used.

About (2), besides neurodegenerative diseases, cancer may represent yet another very interesting field for exploring antioxidants and prooxidants as therapeutic substances due to their cytotoxic effects (including overproduction of ROS) that, if achieving proper selectivity, may be used for cancer cells destruction (Fang et al. [18]). To achieve this goal, a unique therapeutic strategy was developed, named as “oxidation therapy,” by delivering cytotoxic ROS directly to the solid tumor or alternatively inhibiting the antioxidative enzyme system, such as HO-1 in tumor. This anticancer strategy was examined by use of O_2_^•−^ or H_2_O_2_-generating enzymes (i.e., XO and d-amino acid oxidase [DAO], resp.) and by discovering the inhibitor of HO-1 (i.e., zinc protoporphyrin [ZnPP] and its polymeric derivatives).

While deleterious when present at high concentrations, low concentrations of ROS exhibit beneficial properties needed for controlling physiological cellular processes (reviewed in Valko et al., 2007 [19]).

Jimenez-Del-Rio and Velez-Pardo [20] have discussed oxidative stress as an important etiopathogenic factor for occurrence and development of neurodegenerative diseases (notably Alzheimer's disease and Parkinson's disease) and cancer. As an extension, possible preventive and therapeutic values of antioxidants were also discussed. Indeed, if considered within a narrow context of oxidative homeostasis, antioxidants may seem to be ideal weapon in preventing and fighting these diseases. However, the context of human pathology is very broad and, so far, there was little benefit of exogenous antioxidants in human intervention studies or clinical trials. There are numerous reasons for these failures. Maybe, the most important one is the design of the preclinical studies, especially related to concentration of the antioxidant used and time parameters relevant to the clinical setting (Kamat et al., 2008 [21]). The imbalance between uncritical acceptance of antioxidants as powerful “drugs” for various pathological conditions and disappointing results obtained in clinical studies has made a sort of confusion. This issue was addressed by Bast and Haenen [16] through listing ten misconceptions related to commercialized applications of antioxidants: (a) “pros”: (1) antioxidants can cure any disease; (2) the more the better; (3) any AO will do (the trick); (4) AO status measures the level of health; (5) natural AOs are superior (over synthesized ones) and (b) “contras”: (1) AOs increase mortality; (2) when present at high doses, antioxidants become prooxidant; (3) theoretically, antioxidants cannot behave as such; (4) once used, antioxidants are inactive; (5) antioxidant drugs do not work.

The first three “pros” clearly cross the line of realistic way of thinking and cannot be considered seriously. The “pro” #4 was very informatively discussed by Pompella et al. [22] who comprehensively presented current problems with the methods (ORAC, oxygen radical absorbance capacity; ferric-reducing ability of plasma; and TEAC, Trolox equivalent antioxidant capacity) routinely used for measurement of total antioxidant capacity (TAC) in plasma (Pompella et al., 2014 [22]). These include lack of needed specificity, especially relevant for ORAC related measurements. Instead, precise measurement of specific compounds is recommended. Regarding the “pro” #5, the situation does not seem entirely clear, as some published metastudies related to protective role of vitamin C in coronary heart disease showed some contradictions (better protection with dietary vitamin C versus synthetic vitamin C) (Ye and Song [23]; Knekt et al. [24]). In any event, this kind of research is anything but simple, as observed health effects of fruit and vegetable ingestion are certainly related not only to the content of vitamin C but also to other macro- and micronutrients and phytochemicals, proven to confer additional health benefits (Carr and Vissers [25]). Similar to “pros,” stated “contras” seem to be a common misconcept related to the design of the study (this is especially relevant for epidemiological studies), relevance of a specific pathological condition and measurement of its outcomes, and, finally, complexity of a living organism. For all these reasons, there is the realistic need for well-designed epidemiological, clinical, and molecular studies that would offer firm evidence and undoubtful conclusions on the role of antioxidants on human health (see also Sections 3.8 and 3.9).

There are still unanswered questions related to oxidative stress and its mediators in pathogenesis of OS-associated diseases. However, it is clear that overproduction of ROS has harmful cellular effects. For that reason, small synthetic antioxidants, molecular scavengers, have been developed to be used in various pathological conditions. The first one, implemented in the clinic for acute brain infarction, was 3-methyl-1-phenyl-2-pyrazolin-5-one (MCI-186, Edaravone, Radicut, norphenazone), approved until now only in Japan (Tabrizchi, 2000 [26]). So far, its free radical scavenging properties were revealed by various biological effects (antioxidant, attenuation of cytokine production, antiapoptotic, antinecrotic, and some other effects), as recently reviewed (Kikuchi et al. [27]).

## 3. 1,4-Dihydropyridines: A Separate Group of Bioantioxidants

1,4-Dihydropyridines could be used as model compounds for studying molecular mechanisms of action modulated by cellular enzymes NADH and NAD(P)H due to their structural analogy to 1,4-dihydronicotinamide [28]. This structure represents the active part of these reduced coenzymes, which are important modulators of various enzymatic redox reactions and are involved in electron transfer.

**Figure 1:**
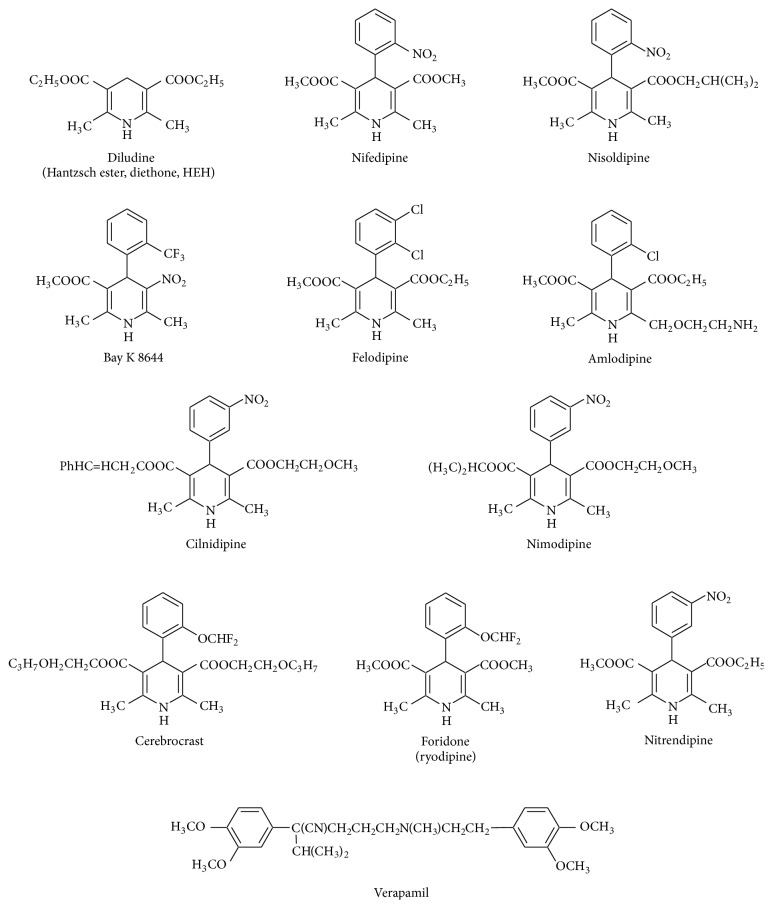
Structures of the most known 1,4-dihydropyridine derivatives and some non-DHP Ca^2+^ antagonists.

**Figure 2:**
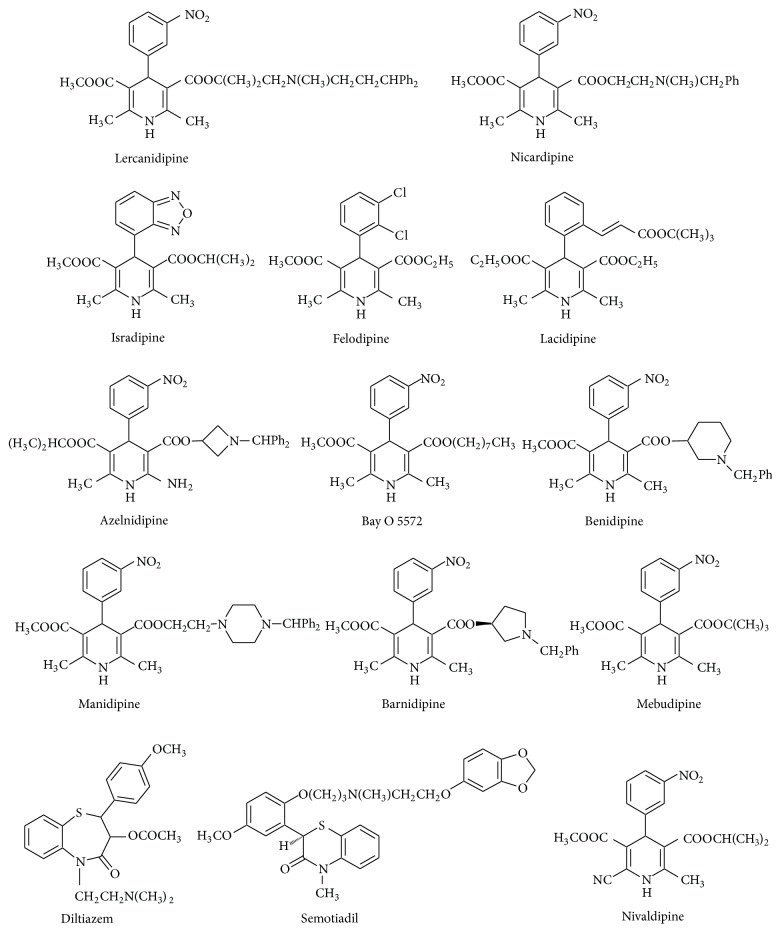
Structures of the most known 1,4-dihydropyridine derivatives and some non-DHP Ca^2+^ antagonists.

**Figure 3:**
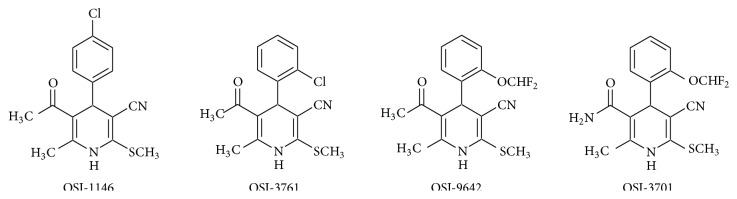
Molecular structures of OSI-1146, OSI-3701, OSI-3761, and OSI-9642 (according to [146]).

Chemically, 1,4-dihydropyridines are synthetic hydrogenated N-heteroaromatic compounds. They may have various substituents at positions 2,6-, 3,5-, and 1,4- (Figures 1–3). Their derivatives can be obtained synthetically in the Hantzsch type cyclic condensation reactions.

Bossert and Vater [29] postulated DHPs as a basis for development of new cardiovascular drugs. Today there are many marketed drugs which contain 1,4-DHP ring as basic scaffold [30–32] (Figures 1 and 2).

Grover et al. [33] classified dihydropyridine nucleus (skeleton) as a novel pharmacophore and offered some examples related to DHPs pleiotropy. So far, AOAs have been revealed for several groups of DHP compounds and DHP-based drugs [34–36], contributing to their well-known pleiotropic ways of action (antiaging, neuroprotective, anticancer, antibacterial, [37] and many more). These features are promising for development of novel drugs in the future [32, 38].

It is well known that hydrogen donors such as amines, thiols (aminothiols), or phenols (plant phenols and polyphenols as well as synthetic hindered phenols) act as antioxidants, primarily through inhibition of oxidation reactions of various chemical targets/substrates. Similarly, depending on their particular chemical structure, 1,4-dihydropyridines have significant hydrogen donor ability (see further in Section 3.2). This feature allows them to act as direct inhibitors of free radical reactions. It further classifies them as specific group of dihydropyridine type of antioxidants. However, under certain conditions, primarily dependent on individual structure and applied dose, DHPs can act as prooxidants (see further in Section 3.8).

On the other hand, some DHPs may exert synergistic effects when applied together with other types of AOs [39]. They can also be involved in the redox regulation of Ca^2+^ ion channels [40]. Namely, oxidative stress, characterized by significant increase of ROS, closely relates to cellular imbalance of Ca^2+^ ions. Such a CA activity of DHPs can also result in the indirect OS modulation as an additional positive side effect. Accordingly, DHPs, acting as CA and as antioxidants, may modify various OS-associated pathological processes by influencing cellular redox signaling potential. Additionally, multiple biological effects of DHPs attenuating OS could be important at drug-drug interactions by combination therapy using DHPs and other CA and/or antioxidants.

It should be mentioned that the studies on the possible AOA of 1,4-DHPs have begun due to the assumption that these substances could be useful for the design for novel antioxidants intended to be used primarily in the food technology, notably as animal chow stabilizers [41–43]. The AOAs of 1,4-dihydropyridine derivatives, 2,6-dimethyl-3,5-diethoxycarbonyl-1,4-dihydropyridine (Hantzsch ester (HEH), diludine) and its close analogues, 4-unsubstituted 1,4-DHPs, were discovered by Latvian scientists that intended to use them for the termination of the lipid peroxidation (LPO) in various chemical lipid substrates/mixtures target (solutions, emulsions, and liposomes) [44, 45]. Afterwards, antioxidant properties of several calcium antagonists DHPs were discovered [31, 46–53]. Interestingly, research on the AOA of DHPs on LPO continues nowadays, including several interdisciplinary projects funded by EU, in particular the COST B35 action [51, 52].

### 3.1. Synthesis of 1,4-Dihydropyridines: Routes and Approaches

Classical 3-component Hantzsch synthesis of DHP compounds [54–57] is usually performed in solutions (including ionic liquids) by heating. Discoveries related to this process and published between 1986 and 1990 are summarized in the review of Sausins and Duburs [57]. In 1993, Kazda [58] has reviewed “twenty years of dihydropyridines,” including their synthesis, chemistry, progress in pharmacology, and therapy, and some other applications. Since then, there were many important discoveries in this field and there is a time for a review on “another twenty years of DHPs.” It has to be mentioned that nearby this classical multicomponent synthesis also a process to obtain structurally diversified 1,4-dihydropyridines at sophisticated conditions was recently reviewed by Wan and Liu [59].

Many discoveries relevant for novel routes in DHP designing and synthesis were published and deposited in various databases (see http://www.organic-chemistry.org/namedreactions/hantzsch-dihydropyridine-synthesis.shtm [60]). For example, http://www.scifinder.com/ [1] database lists approximately 1000 citations on the simple DHP compound, diludine.* Reaxys* database [61] contains data related to variations in starting materials, intermediates as building blocks, media, and reactions routes. Water and ionic liquids as reaction media, microwave and infrared irradiation, new catalysts, solid phase synthesis, and biotechnology based and green chemistry approaches were also proposed as attractive options for syntheses of DHPs [62–66].

Furthermore, several new dihydropyrimidin-(2H)-ones (DHPMs), close analogues of DHPs, were prepared in the Biginelli reaction under ultrasound irradiation and in the presence of NH_4_Cl. Some of these compounds, when tested* in vitro* at concentrations higher than 100 *μ*M [67], showed AOAs, manifested as inhibition of LPO induced by complex Fe + EDTA and reduction of ROS levels.

Recently, Sun et al. [68] reported about the synthesis and antioxidant activity of a series of novel 3-chalcone-substituted 1,4-dihydropyridine derivatives, based on dimethyl or diethyl 2,6-dimethyl-4-phenyl-1,4-DHP-3,5-dicarboxylate.

### 3.2. 1,4-Dihydropyridines as Hydrogen Donors

Steric, electrostatic, and hydrophobic descriptors in DHP molecule could serve as its potential pharmacophores [2]. In case of Hantzsch ester this implies partly hydrogenated N-heteroaromatic DHP nucleus itself or its fragments, that is, NH group or C-4 H- atom, as hydrogen donors necessary for the AO activity and/or carboxylic ester side groups (its C=O group and O atom as hydrogen bond acceptors) in positions 3- and 5- and alkyl side groups in positions 2- and 6- (as hydrophobic features) (Grover et al. [33] and Tikhonov and Zhorov [69]). The presence of labile hydrogen atoms (mainly in positions 1,4-) in DHPs molecule assigns significant hydrogen donating ability to these compounds.

**Scheme 1:**
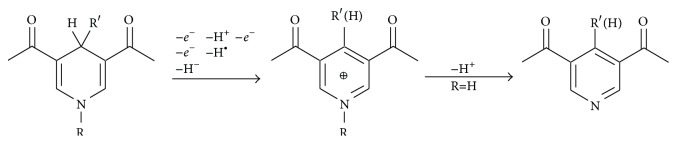
Reactions of 1,4-dihydropyridines leading to the formation of pyridine derivatives.

DHPs (2,6-dimethyl-1,4-dihydropyridine-3,5-dicarboxylic acid esters) can be oxidized in chemical (Dubur and Uldrikis [70]), electrochemical, enzymatic (Duburs et al. [71]), and biological (including metabolism and biotransformation) systems. As already stated, dihydropyridines (especially unsubstituted in position 4) may transfer the hydrogen, similar to the reduced diphosphopyridine nucleotides, NADH and NADPH (Scheme 1) (Mauzerall and Westheimer [28]), while HEH hydrogen transfer studies and search for novel NADH model compounds are continuously developing (Xie et al. [72]).

Tamagaki et al. [73] observed metal-ion-facilitated oxidations of DHPs with molecular oxygen and hydrogen peroxide. On the other side, Tirzite et al. [74] studied some 1,4-DHP derivatives as reductants in relation to trivalent iron. Hantzsch esters have been extensively utilized as stoichiometric biomimetic reducing agents. Recent summarized literature about DHPs as reducing agents, including references on diludine, may be found on specialized websites: http://www.organic-chemistry.org/chemicals/reductions/ [75].

DHPs form free radicals in chemical, electrochemical, and biological oxidation processes. The kinetic parameters and pathways of decay of the cationic radicals formed as primary products in the course of electrooxidation of the esters of 1,2- and 1,4-dihydropyridine have been extensively studied [76].

The regenerative system of nicotinamide cofactors may involve oxidizing or reducing reagents, regulating enzymes, and photochemical reactions. Thus,* in situ* regeneration of the consumed cofactors was observed in the biosystems engineering, which create superior biocatalysts by the reduction of NAD(P)^+^, which can lead to the 1,4-DHP product (which is the only active form) and to the 1,6-DHP compound [77]. The NADPH models of HEHs can be regenerated* in situ* as biomimetic hydrogen sources by means of transition metal/Brønsted acid catalyzed relay asymmetric hydrogenation [78]. General regeneration strategies were reviewed by Chenault and Whitesides [79]. Based on these strategies, particularly related to methods of preparation and practical use of esters of 2,6-dimethyl-1,4-dihydropyridine-3,5-dicarboxylic acid as antioxidants that might be probably applicable for radioprotection and adjuvant treatment against metastases, several patents were prepared [80].

Sambongi et al. [81] have found that the novel water-soluble Hantzsch 1,4-dihydropyridine compound (the potassium salt of 2,6-dimethyl-1,4-dihydropyridine-3,5-dicarboxylic acid monomethyl ester) functions in biological processes through regeneration of NADH. Various parameters related to nicotinamide coenzymes regeneration, especially in a light of chiral compounds, have been published recently [82], while Okamura et al. [83] reported the use of the oxidative conversion of dihydropyridine to pyridinium ion and the metabolic trapping principle as an approach for measuring* in vivo* cerebral redox states.

### 3.3. Antioxidant Activity (AOA) and Antiradical Activity (ARA) of 1,4-Dihydropyridines

Antioxidative activity of 1,4-DHPs was first evaluated and studied in the Latvian IOS (Tirzit and Duburs [39], Zilber et al. [44], and Dubur et al. [45]).

In the field of ARA, pioneering work was made by Schellenberg and Westheimer [84] in 1965. In 1979, Schellenberg [85] revealed the free radical oxidation of a dihydropyridine following Huyser et al. [86] who reported hydroxyl radical quenching by DHPs, especially Hantzsch ester, studying free radical oxidation of DHPs* in vitro*, in the Fenton system.

AOA and ARA of various 1,4-DHPs were further studied by several different methods in both* in vitro* and* ex vivo/in vivo* systems [3, 31, 44–53]. CA nisoldipine, nimodipine, nitrendipine, nifedipine, and nicardipine have AOA that correlates with their lipophilicity (modified Buege and Aust's method of TBA determination, applied in model of rat brain cortex ischemia/reperfusion) [87].

N-Aryl-DHPs, designed as sirtuin activators, were further reported as suitable agents for neuroprotection due to their radical avoidance properties (Hardeland [88]).

1,4-DHPs inhibit free radicals and, consequentially, the cascade of events related to lipid peroxidation. They may influence several stages (initiation and/or propagation) of the lipid peroxidation cascade, which consist of ~10 reactions [89] (detailed discussion in Section 3.3.1 (2)-(b), Scheme 2).

However, considering the great number of AO compounds (including DHPs) and the diversity in their action mechanisms [90],* in vivo* studies are not always convincing and conclusive. Therefore, concise* in vitro* models are necessary to screen each compound with antioxidative properties. Antioxidants are designed to react readily with oxidizing species and are often extensively oxidized already during incubations at atmospheric oxygen tension (oxidation of some water-soluble DHPs in water (buffer) solutions is very fast, especially in the presence of light). Even during a relatively short incubation period, the concentration can drop drastically, and the real potency of the compound could be underestimated [90].

#### 3.3.1. Common AOA and ARA Features of Some DHPs

*(1) In Vitro (in Solutions, Emulsions, and Liposomes)*. Basic molecular principles related to antioxidative and antiradical activity of various antioxidants, including DHPs, were published recently [91]. These data show that DHPs react with various types of free radical species, stable free radicals (DPPH a.o.) and alkyl radicals and with oxygen and nitrogen free radicals. Some derivatives of DHPs may quench a singlet oxygen and may react with peroxynitrite anion [92–95].

Reactivity of DHPs toward alkyl radicals was studied electrochemically [96].

The activity against DPPH radical was found for the 5-acetyl-2-alkylthio-4-aryl-6-methyl-1,4-dihydropyridine-3-carboxylic acid nitriles [97], structural analogues of the 5-acetyl(carbamoyl)-6-methylsulfanyl-1,4-DHP-carbonitrile (studied as mitochondriotropic compounds; see further in the text, Section 3.3.1 (2)-(b)). The highest antiradical activity occurred for a compound which contains two hydroxyl groups in the 4-phenyl substituent.

DHPs were proved to decrease oxygen uptake (2-3-fold) in the heme (methemoglobin, hematin, hemin, and cytochrome C) catalysis by oxidation of emulsions of esters of unsaturated fatty acids and liposomes of phospholipid phosphatidylcholine (Zilber et al. [44] and Dubur et al. [45]).

Reactivity of nitrosoaryl and nitroaryl derivatives of 1,4-DHPs toward alkyl, alkylperoxyl radicals, and ABTS radical cation was found in various LP modeling systems suitable for determination of DHPs AOA and ARE features [98–104]. Diludine and foridone and its analogues were shown to inhibit lipid peroxidation through inhibitory effect on lipoxygenase, in emulsions and in reversed micelles (Tsetlin et al. [105] and Panek et al. [106]). In addition to inhibition of thermally initiated oxidation of methyloleate in the solution [107] (where AOA of 4-unsubstituted 3,5-dicarbonylderivatives of 2,6-dimethyl-1,4-DHPs is not linearly dependent on the inhibitor concentration), DHPs derivatives containing hydroxy, alkoxy, or dimethylaminophenyl substituents in position 4 were shown to prevent loss of *β*-carotene in the disperse system of *β*-carotene and methyllinoleate (Plotniece et al. [108]).

The AOA of DHPs has been detected using different methods in various systems where lipid free radical generation (nonenzymatic, Fe^2+^-dependent, and/or enzymatic, NADPH-dependent) took the place [109–112]. This activity was further confirmed* in vivo*, through prevention of damage caused by renal ischemia and reperfusion, as shown for diludine [113].

Some redox properties of calcium antagonist dihydropyridines were revealed through electroanalytical studies [114]. Competitive kinetic procedure was used for exploring the AO capacity of five (four 1,4-DHPs: lacidipine, felodipine, nifedipine, and amlodipine, and one 1,2-DHP compound GR44966) CA and one calcium ion agonist (Bay K 8644). All but one (amlodipine) antagonist displayed an unambiguous AO capacity (crocin test). The calcium agonist DHP revealed no reaction with peroxyl radicals. Lacidipine was the most effective. A calcium agonist Bay K 8644 is quite resistant to oxidation and does not bind H^+^. This could be important fact in the interaction with the target proteins (it should be mentioned that there are no studies on LP with other Ca^2+^ agonists).

The decreased oxidation potential correlates with AO capacity and increased basic character. These findings suggest the relevance of the electron density on the DHP ring.

For all the DHP compounds investigated, the overall oxidation process proceeds through two consecutive one-electron releases: a primary one-electron step accompanied by a fast proton release and the formation of a neutral radical (PyH^∙^) undergoing a second, much easier one-electron step [114].

The final product is the protonated form of the parent pyridine derivative. This pattern is relevant for the antioxidative activity, since the radical intermediate is far less prone to be reduced than oxidized.

In the case of CA DHPs, the release of protons complicates the overall oxidation process by introducing a “parasitic” side reaction where a coupling between protons and the starting species takes place.

This DHP self-protonation subtracts part of the original species from the electrode process because the parent cationic species are no longer electroactive.

Conversely, the calcium agonist DHP, which is less prone to be oxidized, turned out to be so weak base to be even unable to undergo the self-protonation reaction.

Thus, the combined effect of oxidation potentials and proton binding capacity of DHPs is a key element for the redox transition, relevant for their AO activity. Yet, opposing effects (antagonistic* versus* agonistic) on protein targets as calcium ion channels connected with protein thiol oxidation to disulfide should be also considered [114].

Kouřimská et al. [115] found AO effect of diludine (HEH) in edible oil. Reactivity of 1,4-DHPs toward SIN-1-derived peroxynitrite was shown by López-Alarcón et al. [116]. Olek et al. [117] discovered antioxidative activity of NADH and its analogue* in vitro*.

Further see, as referred in several subparts below, Sections 3.4, 3.5, and 3.7.

*(2) Ex Vivo (on Lipid Peroxidation in LDL, Mitochondria, Microsomes, and Cells)*. Main chemical structures of DHPs examined in numerous studies and reviewed in this paper are presented in Figures 1 and 2.

*(a) Various DHPs: Calcium Antagonists as Inhibitors of LDL Peroxidation*. Free radicals induce peroxidation of LDL. This process proceeds by a chain mechanism which reveals phosphatidylcholine hydroperoxides and cholesteryl ester hydroperoxides as the major primary products [118]. Calcium antagonist DHPs could act as antioxidants on LDL at least in three ways: (1) as inhibitors of isolated LDL peroxidation, caused by various inducers (Cu^2+^ ions, UV light, and xanthine/xanthine oxidase system); (2) if preincubated with cells, preventing against intracellular LDL oxidation; (3) preventing against the harmful effect of oxidized LDL on cells and decreasing cytotoxicity [119–131].

Combined application of ascorbic acid and CA DHPs (amlodipine and felodipine) has an additive (cytoprotective and LDL antioxidant activity) effect [120]. It includes a combination of peroxide-degrading and peroxyl radical scavenging reactions, thus demonstrating the importance of LP during LDL oxidation and cytotoxicity induced by oxidized LDL. Cytoprotection is associated with inhibition of oxidant-induced increases in intracellular free calcium.

Similar to the other model systems, the recorded values of the tested DHPs related to AO activity on LDL LP and related events [119–131] depend on the prooxidant model system and methods used for activity measuring (see Tables 1–5).

Commercial Ca^2+^ antagonists (including 1,4-DHP derivatives), as well as some other 1,4-DHPs with less CA activity, were shown to decrease the rate of oxidation (detected as TBARS) of low-density lipoprotein (LDL) induced by Cu^2+^ ions (CuSO_4_) in two different cell lines: U937 human monocyte-like and J774A.1 murine monocyte-macrophage cell line (Rojstaczer and Triggle [119]). The strongest effect was recorded for vitamin E, followed by felodipine, 2-Cl analogue of nifedipine, nifedipine, amlodipine, nitrendipine, verapamil, and diltiazem.

Rojstaczer and Triggle [119] found that CA from different chemical groups had a concentration-dependent effect as antioxidants against LDL oxidation (see Table 1). However, the order of potency (activity rank order, ARO) of the drug(s) again depends on the oxidation system and the antioxidant assay. Both CA and antioxidative effects relate to the 2- (or* o*-,* orto*-) substituent of the 4-phenyl ring in the same potency order *o* > *m* ≫ *p* [119]. On the other hand, the requirement for the 1,4-DHP ring is essential for both AOA and Ca^2+^ channel antagonism. A charged substituent at the position C-2 of the 1,4-DHP ring influences the AO activity (analogous to [46–53]). However, some other factors should not be neglected: for example, although amlodipine has a positively charged amine at this position, this modification makes it less lipophilic and, indirectly, less potent antioxidant.

Similar results were obtained when testing antioxidant effect of CA on LDL peroxidation in bovine aortic endothelial cells (BAECs) (Cominacini et al. [123]; see Tables 2 and 3) as well as in HUVECs (Lupo et al. [129]) (see Table 4).

Cominacini et al. [123] observed antioxidant effect of CCBs and *α*-tocopherol in BAECs. The order of potency (see Tables 2 and 3) [123] was however different than in U937 human monocyte-like and J774A.1 murine monocyte-macrophage cells (see Rojstaczer and Triggle [119], Table 1). The tested DHPs were lacidipine, amlodipine, lercanidipine, nimodipine, and nifedipine (in two different intracellular concentrations: 2 and 4 fmol). ROS production was significantly lowered only by lacidipine (which is the compound with the highest lipophilicity) and lercanidipine; the effect of lacidipine was much more evident than lercanidipine. Surprisingly, amlodipine, nimodipine, and nifedipine had no effect on ROS formation suggesting that the positive effects on the earliest events of atherosclerosis are a peculiarity of lacidipine molecule through its antioxidant activity.

The strong AO action of lacidipine may be related to the lipophilic cinnamic acid side chain, which favors a drug partitioning in the membrane due to favorable physicochemical (hydrophobic) interactions of drug hydrophobic residues with polyunsaturated groups of membrane phospholipids. However, DHPs can also reduce the oxLDL-induced ROS concentration by affecting some intracellular ROS producers, such as NADPH oxidases, xanthine oxidase, and cyclooxygenase enzymes. The activity of these enzymes contributes to intracellular ROS elevation [125].

Preincubation of HUVECs with lacidipine inhibited an increase of intracellular ROS caused by oxidized LDL [124].

Lupo et al. [129] have studied the dose-dependent (1, 5, 10, and 50 *μ*M) AOA of various CA (verapamil, diltiazem, and DHPs: nifedipine, amlodipine, isradipine, or lacidipine) against normolipidemic human blood LDL oxidation compared with *α*-tocopherol by measuring the content of TBARS and the diene formation (see Table 4).

As presented (Table 4, according to [129]), for diltiazem (poor lipid solubility), no AO was detected, whereas the other CA and *α*-tocopherol have demonstrated AOA at least at concentrations of 10 and 50 *μ*M: *α*-tocopherol > lacidipine > nifedipine > isradipine, verapamil, and amlodipine. Additionally, *α*-tocopherol and lacidipine were able to significantly attenuate* in vitro* LDL oxidation at 1 and 5 *μ*M. These results have confirmed the highest activity for the strongly lipophilic DHP type CA compound lacidipine. This might be a possible antiatherogenic mechanism of CA, since oxidative modification enhances the atherogenic potential of LDL.

The lipid peroxidation of LDL, promoted either by UV radiation or by copper ions, was inhibited (antioxidant effect) by nisoldipine in a dose-dependent manner (IC_50_ values were evaluated at around 10 *μ*M), nimodipine was less potent (IC_50_ around 50–100 *μ*M) and nicardipine almost inactive. In addition to this indirect protective effect, CA DHPs nisoldipine and nimodipine exerted direct protective effect on lymphoid cells, against toxicity of previously oxidized LDL. The IC_50_ values were 6 ± 2 and 80 ± 20 *μ*M, respectively [122]. The inhibition of the cytotoxic effect of LDL oxidized in the presence of DHP type Ca^2+^ channel blockers correlated well with protection from oxidation by these compounds. Complete protection cannot be obtained because the DHPs are cytotoxic themselves. The potential relevance to the prevention of atherogenesis is envisaged.

DHP type CCB nifedipine was the most effective inhibitor of oxidation promoted either by UV radiation or by copper ions in experiments with cultured lymphoid cells LDL (2 mg apoB/mL); CCBs from other two CCB classes, diltiazem and verapamil, were only poorly active or completely ineffective [121]. The protective effect of nifedipine occurs at two levels: besides its direct antioxidant effect by inhibition of LDL oxidation, it also exhibits a direct cytoprotective effect against cytotoxicity of oxidized LDL by yet unknown mechanisms. The protective effect of CCBs was not due to an inhibition of LDL uptake. This effect seems to be independent of the inhibition of LDL oxidation* per se* since LDL was oxidized in the absence of the drug before the incubation with cells. Moreover, this direct protective effect was observed at lower concentrations (IC_50_ of 1 ± 0.2 *μ*M) compared to the antioxidant effect (IC_50_ of TBARS inhibition is around 10 ± 2 *μ*M at UV promoted and 4 ± 0.5 *μ*M by Cu^2+^ ions initiated). The AO effect of nifedipine is also correlated with the protection of endogenous tocopherols (IC_50_ = 50 *μ*M). It was suggested that the AO effect of CCBs protected cells indirectly from the cytotoxic effect of oxidized LDL [121].

A recent study has reported that beneficial vascular effects of lercanidipine in diabetic rats depend on its antioxidant activity related to attenuating the increase in oxidative stress and in vascular matrix metalloproteinase-2 (MMP-2) (Martinez et al. [126]). Lesnik et al. [127] studied the impact of a combination of this calcium antagonist and a *β*-blocker atenolol on cell- and copper-mediated oxidation of LDL and on the accumulation and efflux of cholesterol in human macrophages and murine J774 cells. They realized that lercanidipine reduced the oxidative modification of LDL rather than diminished cholesterol accumulation in human foam cells.

Comparing the antioxidative action of CA (DHPs, amlodipine, lacidipine, nifedipine, and isradipine, as well as diltiazem and semotiadil) in the copper-catalyzed oxidation of low-density lipoprotein (LDL) with that of glycated (g)/glycoxidated (go) LDL demonstrated that the strongest AO effects during long-term LDL glycation are seen for isradipine, lacidipine, nifedipine, and semotiadil [128]. Inhibitory effects were in the range 10^−5^–10^−3 ^M. Authors suggested that, due to the increased generation of ROS by glucose-modified LDL, the chain-breaking capacity of CA may be overridden. The AOA of CA depends on their lipophilicity and their ability to incorporate into the LDL particle, that is, to reach the site of peroxidation. CA, like other AOs, significantly retards advanced glycation end products (AGE) formation, whereas initial glycation reactions, such as Amadori product formation, are only weakly inhibited. The observation that both oxidative changes and at least long-term glycation effects are indeed drastically reduced by CA is corroborated by fluorescence analysis, AGE-ELISA, quantitation of lipid peroxidation, and TBARS measurement of long-term g/go LDL.

The effects of lipophilic DHP calcium channel blockers on oxidized LDL-induced proliferation and oxidative stress of vascular smooth muscle cells were also studied [130] (see Table 5).

Lacidipine and amlodipine reduced carotid intima-media thickness by decreasing proliferative effect of oxLDL, whereas (*S*-)-amlodipine had no antiproliferative effect. ROS-MAPKs (mitogen-activated protein kinases) pathway might be involved in the mechanism.

Both 1,4-DHP CCBs lacidipine and nifedipine reduce plasma and LDL oxidation and formation of oxidation-specific epitopes. Their application may also relate to prolonged survival of rats, independently of blood pressure modifications (in the SPSHR model, 1 mg/kg per day lacidipine and 80 mg/kg per day nifedipine). These results suggested that the protective effect of these two 1,4-DHP drugs* in vivo*, as shown in cerebral ischemia and stroke, may in part result from inhibition of LDL oxidative process, although these two drugs possess different lipophilic properties [131]. Both lacidipine (0.3 and 1.0 mg/kg) and nifedipine (80 mg/kg) prolonged lag time of the conjugated diene formation in LDL isolated from arterial wall, and *t*_max_. These drugs significantly reduced electrophoretic mobility of oxLDL from SPSHR subjected to X/XO oxidation system. 1,4-DHP CCBs also protected apolipoprotein B, which is important for the binding with macrophage LDL receptor lysine residues. The doses used (>10^−6 ^mol/L for SPSHR and normotensive WKY rats), however, are 2 to 3 orders of magnitude higher than those inhibiting vascular smooth muscle contraction* in vitro* and* in vivo*. They also exceed values that are commonly used in clinical practice. The daily dose of lacidipine for hypertensive patients is 0.07 mg/kg, ≈4- to 14-fold lower than the 2 doses used in SPSHR. The maximum daily dose of nifedipine given to hypertensive patients is 2.0 mg/kg, ≈40-fold lower than what were used [131]. These discrepancies may be related to differences in bioavailability of CA between rats and humans [131].

Accordingly, in routine clinical use, 1,4-DHP CCBs do not reach the concentrations required for antioxidant activity* in vitro* [131].

Another data concerning the effect of CA DHPs on OS related to LDL is presented under Section 3.5.

*(b) Effect of DHPs on Isolated Rat Liver and Heart Mitochondria*. As a major cellular source of oxygen radicals (Cadenas [4, 5]), mitochondria are promising targets for pharmacological and toxicological actions of various membrane-active compounds, including several 1,4-DHP derivatives. Zernig et al. [132] have discovered CA binding sites associated with an inner mitochondrial membrane anion channel.

More than 40-year long research on mitochondrial effects of the DHPs (on their bioenergetics, chemiosmotic properties, and ion fluxes) clearly points them out as mitochondriotropic compounds.

The activity of the first 35 synthesized compounds (derivatives of 1,4-DHP, their heteroaromatic analogues, NAD-H^+^ and butylated hydroxytoluene (BHT, BOT)) originally was examined in rat liver mitochondrial LP system, in the presence of Fe^2+^ ions and using the ultraweak chemiluminescence method (Dubur et al. [89]).

Several 1,4-DHP derivatives, Hantzsch ester diludine and its analogues, were found to be effective antioxidants in this experimental system, changing the kinetics of LP, lengthening the time of the appearance of the maximum of the slow burst of the chemiluminescence (latency, latent period), and diminishing the reaction rate (the tangent of the slope angle during the time in which the amplitude of the slow burst characterizing LP rate increases) and its peak value. Their presence has influenced the reaction constant *K*_6_, in relation to a very significant reduction of lipid hydroperoxides and/or inactivation of free radicals, as follows:

(1) $$\begin{array}{c} {ROO}^{•}+{ROO}^{•}⟶P+h\nu^{*}\text{ termination} \end{array}$$

(P = molecular products) or

(2) $$\begin{array}{c} {ROO}^{•}+{ROO}^{•}+H_{2}O⟶ROH+{RO}^{•}+{\;}^{1}{O_{2}}^{*} \end{array}$$

In this study, diludine was one of the most active compounds. DHPs had activity similar to the standard synthetic AO-BHT (ionol). However, when plotted against applied concentration and time window, diludine's activity profile differed from that of BHT.

There were also similar studies (using different LP rate experimental detection system and method, Hunter et al. [133]), based on exploring a group of 26 2,6-dimethyl-3,5-disubstituted- and 2,6-dimethyl-3,4,5-trisubstituted-1,4-dihydropyridines (1,4-H_2_Py=1,4-DHPs) and five related pyridines as inhibitors of rat liver Mit swelling (Δ*A*_520_/*t*) and O_2_ uptake by ascorbic acid- (AsA-) dependent lipid peroxidation and as modulators of Mit swelling induced by Na^+^-linoleate or Na^+^-pyrophosphate (Velēna et al. [112]).

Some of tested 4-DHPs (4-unsubstituted 3,5-dialkoxycarbonyl-2,6-dimethyl-1,4-DHPs and 3,5-diamido-2,6-dimethyl-1,4-DHPs, both 4-unsubstituted, or those possessing lipophilic 4-aryl- groups) have shown significant AO and membrane stabilizing activity. These studies further revealed that 1,4-DHPs preferably act as AO during the stages of initiation and prolongation of LP chain reactions, at low concentrations. The studied 1,4-DHPs had IC_50_ (when *V*_0_/*V* or *τ*/*τ*_0_ = 2) 0.1 *μ*M to 100 *μ*M and the minimal activity was scored for oxidized (heteroaromatized) derivatives.

At the concentration of 100 *μ*M, 3,5-di-*n*-butyloxycarbonyl-2,6-dimethyl-1,4-DHP entirely stops mitochondrial swelling in the presence of 0.8 mM Na^+^-pyrophosphate. At the same concentration, the following compounds alter the mitochondrial swelling rate in the presence of natural protonophore, Na^+^-linoleate: 3,5-di-*p*-hydroxyphenoxycarbonyl- and 3,5-di-*p*-tolyloxycarbonyl-2,6-dimethyl-1,4-DHPs, 3,5-diethoxycarbonyl-2,6-dimethyl-pyridine (oxidized form of Hantzsch ester), and more lipophilic 3,5-diamyloxycarbonyl-2,6-dimethyl-pyridine. The alteration of swelling may be scored as prolonged, promoted, accelerated, or inhibited. The type of alteration depends on the structure and concentration of 1,4-DHPs, the type of initiators of the swelling process, and the medium composition.

In accord with previously published Janero's results (lack of AO for Ca^2+^ antagonists, nifedipine and nicardipine, even at 500 *μ*M concentration in LP tests performed on heart membrane [134]), no antioxidative activity for 4-phenyl substituted derivatives of 3,5-dialkoxycarbonyl 1,4-DHP (close analogues of Ca^2+^ antagonists) was found, contrary to various 4-nitrophenyl 1,4-DHP derivatives, calcium antagonists, for which the significant antioxidant activity was reported [31, 46–53].

Studies made on phosphatidylcholine liposomes (our unpublished data) suggest approximately three and two times more antioxidative activity for 100 *μ*M 4-unsubstituted DHP compound diludine, when compared to 4-substituted DHPs riodipine/nifedipine and nicardipine, respectively, at methemoglobin-induced LP (oxygraphy).

Inhibition of mitochondrial AsA-dependent LP and stabilization of mitochondria were shown to be characteristic for a large group of 1,4-DHP compounds [112], showing to possess the AOA in simplest* in vitro* systems (Tirzit and Duburs [39], Zilber et al. [44], and Dubur et al. [45]) based on reactions with the stable free radical 1,1-diphenyl-2-picrylhydrazyl (DPPH), LP of fatty acid ester (linethole and methyloleate) emulsions, and phospholipid (phosphatidylcholine) liposomes. Generally, these properties did not coincide with Ca^2+^ antagonism. Depending on DHP structure, it seems that AOA properties are less specific than Ca^2+^ antagonist properties. Both properties may be interrelated but not interdependent.

These data show that the presence and the nature of a substituent in position 4, as well as 3,5-substituents, are important factors for 1,4-DHP antioxidant effects in various systems, that is, AsA-dependent nonenzymatic as well as enzymatic NADPH-dependent lipid peroxidation. Sometimes, the efficacy of inhibition of nonenzymatic LP by 1,4-DHPs is higher than the inhibition of the enzymatic LP. However, the action may be opposite, stimulation of the LP. Hantzsch ester (HEH, diludine) and its close analogues exhibited significant AOA and membrane stabilizing properties in both AsA-dependent nonenzymatic peroxidation of mitochondria and NADPH-dependent enzymatic LP of microsomes, usually at similar 10 to 100 *μ*M concentrations [112].

The order of AO potency (IC_50_ values)* in vitro* depends on drug structure as well as on the experimental conditions and specificity of the biological system. Each method for determination of AOA and ARA has advantages and disadvantages (Karadag et al. [135]).

Accordingly, as reported by Gubskiĭ et al. [136], IC_50_ for the AsA-dependent LP was 0.25 *μ*M and 2.0 *μ*M for 1,4-DHP Ca^2+^ antagonists nitrepine (nitrendipine) and nifedipine, respectively. Takei et al.'s [137, 138] studies on mitochondrial swelling induced by LP or arachidonic acid in the rat brain determined the IC_50_ values of 12.7, 10.5, 156.8, and 38.4 *μ*M for efonidipine, nicardipine, nifedipine, and nimodipine, respectively. For LDL in the copper-induced oxidation system the order of potency was vitamin E > felodipine > 2-chlorophenyl analogue of nifedipine > nifedipine > amlodipine, nitrendipine, verapamil, and diltiazem (Rojstaczer and Triggle [119]).

It was interesting to compare the AOA of DHPs with their susceptibility to oxidation, that is, electron and hydrogen donating properties.

It has been estimated that electron donor substituents in positions 2 and 6 of 1,4-DHP cycle usually promote oxidation, while electron acceptor substituents promote quench oxidation. Stronger electron acceptors in positions 3 and 5 also significantly quench oxidation. These estimations are based on studies including chemical, enzymatic, and electrochemical oxidation of 1,4-DHP derivatives (Dubur and Uldrikis [70], Duburs et al. [71], and Stradin et al. [139]).

On the other hand, diminished AOA of 1,4-DHP relates to presence of substituents in position 4 (both electron donor and electron acceptor) (Velēna et al. [112]).

3,5-Dicarbamoyl substituents possess minimal quenching feature and are followed by benzoyl-, acetyl-, and alkoxycarbonyl- groups. Maximal decrease was obtained with condensed substituents (i.e., oxoindeno- or oxocyclohexeno- groups) and a CN-group. 4-Unsubstituted 3,5-dicarbamoyl derivatives can be easily oxidized and consequentially inactivated, whereas 4-substituted 3,5-dicarbamoyl-1,4-DHPs possess an oxidation potential, analogous to the 4-unsubstituted 3,5-COOR derivatives. Therefore, they have adequate electron donor properties and are considerably stable. This may be the reason for significant membrane stabilization upon exposure to 4-substituted derivatives. Of importance, their AOA was usually more pronounced in comparison to 4-unsubstituted derivatives.

Among them, 2,6-dimethyl-3,5-difurfuryloxycarbonyl-1,4-DHP showed the highest antioxidative activity. In the group of 3,5-dialkoxycarbonyl derivatives, the strongest activity was attributed to compounds with medium length alkyl chains (*i*-butyl-,* t*-butyl-, and* i*-amyl- substituents), high level of lipophilicity, minimal electron acceptor properties, and moderate steric hindrance, as contrasted to short or long alkyl chain ester derivatives (3,5-dimethoxycarbonyl-, 3,5-diethoxycarbonyl derivatives and 3,5-didodecyloxycarbonyl derivative). These data demonstrate the bell-shaped dependence of AOA on alkyl chain length [112] and are in accord with results obtained in liposomes. However, these data differ from those obtained in emulsions, where diludine was the most active compound. Finally, oxidized heteroaromatic derivatives showed only minimal activity.

In both LP systems studied (AsA-dependent in mitochondria and NADPH-dependent in microsomes), some of 1,4-DHPs showed activity similar to classical antioxidant, butylated hydroxytoluene (ionol, BHT) (Velēna et al. [112]). However, there was a significant difference related to concentration and incubation time. It allowed us to postulate that 1,4-dihydropyridines (InH), acting as antioxidants-reductants and scavengers of reactive oxygen species and lipid free radicals, preferably influence initiation and propagation (prolongation) of lipid peroxidation chain reactions (1)–(5), according to Scheme 2. The phenomenon is particularly prominent in the presence of Fe^2+^ and other ions of variable valency.

Chain break and termination reactions (6)–(10) of the LP reaction cascade [89] were influenced by 1,4-DHPs in a lesser degree than were initiation and propagation steps. This may be important for their therapeutic effects even in the advanced stages of LP.

**Scheme 2 (stages of initiation, propagation, and termination of lipid peroxidation chain reactions (1–10)).**

Initiation and propagation reactions are as follows:

(1) HOO^•^ + RH →R^•^ + H_2_O_2_ (RH = membrane lipid)
 HO^•^ + RH →R^•^ + H_2_O
 HOO^•^ + InH →In^•^ + H_2_O_2_ (InH = 1,4-DHP)
 HO^•^ + InH →In^•^ + H_2_O
(2) R^•^ + O_2_→ROO^•^ (R^•^; RO^•^; ROO^•^ = lipid radicals)
(3) ROO^•^ + RH → ROOH + R^•^
(4) ROOH + Fe^2+^→RO^•^ + Fe^3+^ + HO^−^
(5) RO^•^ + RH → ROH + R^•^; R^•^ + InH → RH + In^•^

Chain break and termination reactions are as follows:

(6) ROO^•^ + ROO^•^→ P + h*ν* ^*∗*^ (P = molecular products) or ROO^•^ + ROO^•^ + H_2_O → ROH + RO^•^ + ^1^O_2_^*∗*^
(7) ROO^•^ + InH → ROOH + In^•^ (ROOH = membrane lipid peroxides)
(8) RO^•^ + In^•−^→ Y (Y = molecular products)
(9) ROO^•^ + Fe^2+^→ Fe^3+^ + X (X = molecular products)
(10) RO^•^ + RO^•−^→ Y (Y = molecular products)

In the reversible swelling of mitochondria accompanying LP (initiated by mixture of 5 mM GSSG/1 mM GSH), several 1,4-DHPs showed low or no activity, manifested only as a decrease of the swelling amplitude, without a rate decrease. An addition of GSH (4 mM) or ATP to swollen mitochondria caused their contraction in both control and tested system. It may be suggested that 1,4-DHPs, acting as antioxidants in mitochondria, preferably influence LP reactions initiated by ions with variable valency or their complexes with heme type compounds: methemoglobin, hemin, hematin, and so forth (Velēna et al. [112]). If the peroxidation process has a maximal velocity and 50 percent of initial O_2_ were consumed, 1,4-DHPs cannot completely break the chain reactions and prevent subsequent membrane damage: by addition of DHP substance at 10 *μ*M concentration at the moment of 50 percent oxygen consumption, the subsequent oxygen uptake proceeded unchanged. This observation is important for the application of DHPs as inhibitors of initiation and, to a lesser degree, propagation stages of LP chain reactions.

The influence of 1,4-DHPs on Mit swelling is not strictly associated with their own oxidation. There is the possibility that the labilizing (or stabilizing) effect relates to surface activity (connected with substituent lipophilicity) or may be the consequence of complexation with some -OH (or -CH_3_) group sensitive receptors at the mitochondrial membrane. Namely, a bathochromic shift of the absorption band maximum (about 10 nm) was observed in the visible region before swelling. However, after swelling in the presence of Na^+^ linoleate, the spectrum returns to its initial value [112].

Some 1,4-DHPs not only protect mitochondria against swelling caused by AsA-dependent LP, salts of fatty acids* in vitro* [112], but also have beneficial effects on repairing their integrity* in vivo*, after exposure to irradiation, hepatotoxins, ischemia, hypoxia, or hypothermia. Some of them were shown to normalize the process of intracellular reparation and physiological regeneration of ultrastructures. They were also shown to stimulate reparative processes. If pretreated with 1,4-DHPs, irradiated mitochondria will not swell (Ivanov et al. [140, 141]).

Diludine, ionol, and some other AOs, mitochondria protectors, act as anti-ischemic agents. If applied prophylactically* in vivo*, they may prevent ischemic and reperfusion lesions in heart, kidney, and other organs (Bilenko et al. [113]). The effect is dependent on applied dose, timing, and way of application. When added onto the cryoconservation medium for mitochondria preservation, 1,4-DHPs prevented decrease of membrane potential, normalized facilitated respiration, and prevented loss of mitochondrial Na^+^ and Ca^2+^ ions, after thawing ([112], see citation number 36 (Subbota et al., Kharkov, 1984) therein). Diludine was stronger protector, when compared to ionol.

CA drug foridone (riodipine) was shown to possess cardioprotective features, primarily due to is protective effect on mitochondria exposed to OS [142, 143].

Similarly, the DHP water-soluble antiarrhythmic compound glutapyrone inhibits initiation of LP by free radicals in erythrocytes and heart mitochondria. Its cardioprotective effect has been experimentally shown in heart mitochondrial membranes, especially during deep hypothermia (Utno et al. [144]).

Cerebrocrast was effective in several translation models mimicking pathological situations, known to be associated with cellular OS. The potential protective action of 1,4-DHP derivatives (4-substituted compounds: cerebrocrast, gammapyrone, glutapyrone, and 4-unsubstituted drug diethone) has been studied in rat liver, in experimental models relevant for oxidative stress and mitochondrial bioenergetics (Fernandes et al. [145]). When succinate was used as the respiratory substrate, higher concentrations (>25 *μ*M) of cerebrocrast depressed respiratory control ratio (RCR), ADP to oxygen ratio (ADP/O), state 3, and uncoupled respiration rates, transmembrane potential (deltapsi), and the phosphate carrier rate. At the same time, state 4 respiration rate was three times increased. At concentrations lower than 25 *μ*M, cerebrocrast inhibited mitochondrial IMAC and partially prevented Ca^2+^-induced opening of the mitochondrial PTP. Gammapyrone, glutapyrone, and diethone did not induce these phenomena. When applied at concentrations up to 100 *μ*M, cerebrocrast, gammapyrone, and glutapyrone did not affect ADP/Fe^2+^-induced LP of mitochondria in rat liver (as measured by oxygen consumption and TBARS formation). On the other hand, low diethone concentrations (up to 5 *μ*M) inhibited it in a dose-dependent manner. Diethone also prevented against deltapsi dissipation induced by LP initiated by ADP/Fe^2+^. Based on these data, it may be speculated that cerebrocrast (inhibition of the IMAC) and diethone (acting as an AO) may provide effective protection of mitochondria during OS. Cerebrocrast has shown some therapeutic potential for treatment of several pathological conditions related to cellular OS [145].

5-Acetyl(carbamoyl)-6-methylsulfanyl-1,4-DHP-carbonitriles (Figure 3) with minor differences in their molecular structure, displaying antioxidant and antiradical activities* in vitro*, show different biological activities. Namely, 4-*p*-chlorophenyl derivative OSI-1146 displays AO and antiradical activities in cardiovascular OS models, whereas OSI-3701 and OSI-3761 display hepatoprotective activity. Thus, these compounds may be potentially useful for treating several pathological processes, including those associated with OS (Fernandes et al. [146]). However, besides mitochondria, the cellular targets for their pharmacological actions have not been fully investigated [146]. All these compounds increase the susceptibility of Mit to MPT. The most potent is OSI-3701, although it does not affect bioenergetic parameters.

Although all these compounds protected mitochondria against LP induced by the oxidant pair ADP/Fe^2+^, OSI-1146 was shown to be the most potent. Current data point out mitochondria as potential targets for protective and toxic actions of DHPs, suggesting that the potential for their use as therapeutic agents should also take into consideration their toxic effects on mitochondria (Fernandes et al. [146]).

Several structurally different DHP derivatives (antioxidant diludine (diethone), as a 4-unsubstituted DHP, 4-substituted DHPs: CA foridone (bicyclic compound), and the 4-phenyldiethone compound where phenyl group is joined to the DHP in position 4) inhibited the 1-methyl-4-phenylpyridinium iodide (MPP^+^) induced ROS production in cerebellar granule cells (CGC) with a distinct potency order: foridone (2,6-dimethyl-3,5-dimethoxycarbonyl-4-(*o*-difluoromethoxyphenyl)-1,4-dihydropyridine) > 2,6-dimethyl-3,5-diethoxycarbonyl-4-phenyl-1,4-dihydropyridine > diludine. They also reversed the MPP^+^-induced decrease of the mitochondrial membrane potential in the same order (Klimaviciusa et al. [147]). Accordingly, it was postulated that the classical two-ring (bicyclic) structure of DHP derivatives represents an advantage in relation to neuroprotection and ROS defense and is independent on compound's properties related to calcium ions.

Novel adamantane-containing 1,4-DHP compounds (Klimaviciusa et al. [148]) were also found to improve mitochondrial functions (MPP^+^ model) (Klimaviciusa et al. [148]). Klusa et al. [149] have discovered antineurotoxic effects of 1,4-DHP taurine derivative, tauropyrone, recorded as Mit function improvement.

Many 1,4-DHPs, including Ca^2+^ antagonists and AO, modify LP processes and influence mitochondrial function in various organs (liver, heart, kidney, and brain) in a different way and degree. Their beneficial action, oxygen or lipid free radical scavenging, antioxidative effects, binding with or intercalating into phospholipid bilayer, regulation of ion gating, and regulation of mitochondrial permeability transition pores (Tirzit and Duburs [39], Zilber et al. [44], and Dubur et al. [45]), separately or in combination with each other, depends on two strong elements: (1) their individual structure including nature of substituents and their positions and (2) the nature of the biological system. For example, the direction of LP (inhibition of promotion) was shown to depend on structure and concentration of applied 1,4-DHPs as well as stages of chain reactions. Accordingly, mitochondrial swelling may be prolonged (retarded), accelerated (promoted), or inhibited (Velēna et al. [112]).

Therefore, there is a ground for 1,4-dihydropyridines, either Ca^2+^ antagonists or antioxidants, to be nominated as useful tools in development of “mitochondrial drugs” related to the control of OS.

*(c) DHPs as AOs in Endoplasmic Reticulum (Inhibition of NADPH-Dependent LP System): Inhibition of NADPH Oxidase by DHPs*. Elevated level of NADPH oxidase 4- (NOX4-) derived hydrogen peroxide (H_2_O_2_) joined with concomitant decrease of nitric oxide (NO) mediated signaling and reactive oxygen species scavengers are considered to be central factor in molecular pathogenesis of fibrosis (Sampson et al. [150]). Inhibition of microsomal NADPH-dependent LP, with particular focus on NADPH oxidases (NOX1–5 and DUOX1), may be very important for neuro-, cardio-, and hepatoprotection (Velēna et al. [112], Leto and Geiszt [151], Griendling et al. [152], and Chen et al. [153]). Endoplasmic reticulum may be an important target, as this is where 1,4-DHPs could display their antioxidative properties (Velēna et al. [112], Leto and Geiszt [151], Griendling et al. [152], and Chen et al. [153]).

However, the initiation of LP in the NADPH-dependent microsomal system does not appear to involve either superoxide or hydrogen peroxide, since neither SOD nor catalase can inhibit it. On the other hand, reduced iron plays an important role in both the initiation and propagation of NADPH-dependent microsomal lipid peroxidation (Hochstein and Ernster [154] and Repetto et al. [111]).

Many DHPs possess inhibitory activity not only towards AsA-dependent LP in mitochondria but also towards NADPH-dependent LP, as shown in isolated rat liver microsomes (Velēna et al. [112]). This means that these compounds interact with the shared parts (nonenzymatic and enzymatic) of LP pathways.

Microcalorimetry and fluorescent probes procedures were used for studying the interaction of alpha-tocopherol and 1,4-DHPs with endoplasmic reticulum membranes and model systems, human serum albumin, and phospholipid bilayers [155]. Modification of microviscosity of the endoplasmatic reticular membranes depends on localization of antioxidants within the protein structures or phospholipid phase. Increase of membrane structuralization under the influence of 1,4-DHPs blocked their antioxidant action in spontaneous and induced lipid peroxidation.

Inhibition of rat heart and liver microsomal lipid peroxidation by nifedipine was observed [156], while Goncalves et al. [157] found antioxidant effect of calcium antagonists on microsomal membranes isolated from different brain areas.

Nitroaryl-1,4-DHPs are both calcium channel antagonists and antioxidant agents (Letelier et al. [158, 159]), commonly used for treatment of cardiovascular diseases. These drugs must be metabolized through cytochrome P450 oxidative system (NADPH-cytochrome P450 reductase), mainly localized in the hepatic endoplasmic reticulum. Several lipophilic drugs generate OS while being metabolized by this cellular system. Thus, DHP antioxidant properties may prevent the OS associated with hepatic biotransformation of drugs. Various commercial and new nitro-phenyl-DHPs were studied against LP using rat liver microsomes under oxidative stress [159].

Incubation of rat liver microsomes with the 4′-nitro-4-phenyl-1,4-DHP compounds (2,6-dimethyl-4-(4′-nitrophenyl)-1,4-dihydropyridin-3,5-diethyl-dicarboxylate and N-ethyl-2,6-dimethyl-4-(4′-nitrophenyl)-1,4-dihydropyridin-3,5-dimethyl-dicarboxylate) results in an inhibition of LP, the UDPGT (UDP-glucuronyltransferase) oxidative activation, and the microsomal thiol oxidation induced by Fe^3+^/ascorbate, a generator system of ROS. This effect was also produced by nitrofurantoin and naphthalene in the presence of NADPH.

Interestingly, IC_50_ of DHPs obtained from microsomal LP assays decreased to the same extent as the microsomal thiols oxidation provoked by Fe^3+^/ascorbate [159]. Nevertheless, the AO effects of a nitrophenyl-DHP compound, in which hydrogen at position one of the DHP ring was replaced by the ethyl group, were significantly weaker. Authors speculated that DHPs can resemble NADH, transferring one hydrogen atom of 4-position (H^−^) to anion superoxide and another of the 1-position (H^+^) by way of a cationic radical intermediate to generate pyridine derivatives and water [159].

The AO effects of various tested DHP derivatives (*m*- and* p*-NO_2_ phenyl as well as methyl or ethyl and isopropyl-DHP 3,5-dicarboxylate derivatives) were not significantly different. The authors assumed that the -NH- group of the dihydropyridine ring could contribute both to the development of the calcium channel antagonism and to the antioxidative properties of DHPs [159].

Prevention of the membrane LP seemingly depends on the concentration of potential antioxidants, such as vitamin E or even 1,4-DHP in lipids. However, only the differences in synthetic DHPs lipophilicity cannot explain significant variations of DHPs concentration in microsomal membrane and cannot clarify the strength of their antioxidative activity. This work [159] has further demonstrated that 1,4-DHPs may prevent the OS induced by biotransformation of some drugs, for example, antibiotic nitrofurantoin. Simultaneous administration of DHPs and nitrofurantoin may be beneficial in reducing nitrofurantoin side effects.

While most of Ca antagonist 1,4-DHPs are metabolized by CYP3A4 (Guengerich et al. [160]), not all of them are good inhibitors of its activity. Thus, nicardipine, but not nifedipine and nitrendipine, inhibits CYP3A4* in vitro* [53]. Interaction of different DHPs with various types of cytochrome P450 was described by Carosati et al. [53]. It was also reported that DHP class calcium channel blockers reduce the antiplatelet effect of clopidogrel (Park et al. [161]). This implies the mutual interactions of both drugs with CYP3A4.

*(3) In Vivo*. Evaluation of nifedipine effects on* Saccharomyces cerevisiae* was recently published (Asma and Reda [162]). Surprisingly, nifedipine exercised a toxic effect on* Saccharomyces cerevisiae* shown through measuring cellular proliferation, respiratory activity, and the level of some biomarkers (CAT and MDA).

However, majority of data obtained on various animal cells and tissues by other authors show the protective role of DHPs against both LP and oxidative stress [113, 163, 164].

The AOA attributed to many 1,4-DHPs, Ca^2+^ antagonists and other compounds, reflecting on catalytic LDL peroxidation (see Section 3.3.1 (2) and Section 3.5), should encourage their testing for treating cardiovascular diseases and/or alterations of lipid metabolism.

The possibility that 1,4-DHP-based calcium antagonists exert an antiatherosclerotic action (*via* inhibition of LDL oxidation and other mechanisms) has been proved by many experimental data [165] and several clinical trials. Besides antihypertensive effect, nicardipine was shown to possess antioxidative and antielastase activity [165, 166]. These properties may be useful for prevention of inflammatory reaction which is relevant for hypertension pathogenesis.

1,4-DHPs administration inhibits LDL oxidation mediated by oxygen radicals, leading to decreased carotid intimal media thickness and reduced progression of coronary atherosclerosis [130]. It additionally preserves Apo B-100 integrity against ROS. Of importance, antiatherogenic mechanisms differ between animals and humans (primarily in the stage of conversion of aldehydes to carboxylic acids) (Parthasarathy et al. [167]).

For example, furyl-DHP compound (FDP-1, diethyl 2,6-dimethyl-4-(furyl)-1,4-dihydropyridine-3,5-dicarboxylate) was shown to act as an antioxidant (decreasing MDA, GOT, and FFA release of ischemic myocardium and inhibiting Ca-ATPase of erythrocyte membranes), preventing against heart myocardium ischemia-reperfusion injury and arrhythmia, when applied (in rats) at 10 mg/kg (Liu et al. [168]).

Similarly, antioxidative effects of azelnidipine and amlodipine prevented neuronal damage by CCBs, after transient focal ischemia in rats (Lukic-Panin et al. [169]).

Allanore et al. [170] found that both nifedipine and nicardipine significantly decrease the mean level of plasma markers for oxidative stress in patients suffering from systemic sclerosis.

Antioxidants may be considered as promising neuroprotective compounds. Still, while experimental data demonstrate neuroprotective effect* in vitro* and in animal models, clinical evidence is still unsatisfactory and insufficient [171].

*(a) Role of Metabolism of DHPs in Their AOA*. Metabolic pathways and “bioavailability” of the probable AOA compound determine antioxidant activity* in vivo*. Antioxidant metabolites may vary in stability and activity leading to two opposite scenarios: lack or presence of activity, substantially contributing to the overall AOA [172]. Metabolic biotransformation of DHPs includes oxidation (heteroaromatization), side chain ester group cleavage (deesterification), and 4-substituent abstraction a.o. [160]. None of the DHPs metabolites was shown to be more toxic than original, reduced form of the compound. The commonly detected metabolites of the DHPs do not seem to possess the AO activity (with some exceptions as in the case of metabolites of nifedipine and its analogues, including nitrosonifedipine [173, 174]) (see further in Section 3.5). Due to DHPs intrinsic instability, achieving and maintaining an adequate concentration may be problematic both* in vitro* and* in vivo*.

*(b) Role of Concentration and Lipophilicity (Membrane/Water or Lipid/Water Partition Coefficients) of DHPs in Their Action as AOs and Antiradical Compounds*. Antioxidative effects of any antioxidant depend on its concentration at the site of action. This parameter is hardly measurable, especially in two-phase systems, representing one of obstacles in comparison to AOA upon applying various compounds [172]. It is often incorrectly assumed that the concentrations in the aqueous solution and at the site of action are the same. However, even when the concentration in the aqueous phase may be well controlled, the concentration at the site of action in the lipid matrix of the membranes might fluctuate between different test compounds, depending on a difference in lipophilicity [175]. The prevention of the membrane LP also seems to be dependent on the DHP concentration in the lipid matrix (Mason and Trumbore [46]) and its amphiphilicity. For example, AOA of diludine is associated with its lipophilicity and consequential ability to be incorporated into liposomes (Panasenko et al. [176]). It was also found that diludine easily incorporates into the outer monolayer of erythrocyte membranes [176].

Membrane/buffer partition coefficients (lambda) were directly measured in the sarcolemma and sarcoplasmic reticulum membranes for three CA DHPs. The obtained values were in a broad range between 5000 and 150000 (Herbette et al. [177]). These drugs interact primarily with the membrane bilayer component but may also bind to proteins, both nonreceptors and receptors. The intrinsic forward rate constants for DHP binding to sarcolemmal calcium channel receptors were apparently not strongly dependent on their membrane partition coefficients. For example, nimodipine (lambda = 6300) had a forward rate constant of 6.8 ± 0.6 × 10^6^/M/s, whereas the forward rate constant for Bay P 8857 (lambda = 149000) was 1.4 ± 0.8 × 10^7^/M/s. Since these DHPs are highly liposoluble, model calculations for this binding reaction demonstrated that these rates on lipid solubility would probably not be reflected in the experimental forward rate constants. In addition, the intrinsic forward rate constant for nimodipine binding to sarcolemmal calcium channel receptors was found not to be linearly dependent on the viscosity of the buffer medium over a fivefold range. The rate of drug nonspecific binding to nonreceptor protein present in highly purified sarcoplasmic reticulum membranes appears to be extremely fast, at least 10^3^ times faster than specific drug binding to the receptor in the sarcolemma. Authors concluded that partitioning into the lipid bilayer matrix of the sarcolemma could be a general property of CA DHPs and may be a prerequisite for their binding to sarcolemmal membrane receptors (Herbette et al. [177]).

The binding of DHP calcium channel agonists and antagonists (including those with AO properties) to receptors in cardiac sarcolemmal membranes is a complex reaction that may involve an interaction with the lipid bilayer matrix of the sarcolemma (Herbette et al. [178]). Belevitch et al. [179] studied the binding of DHP CCBs (riodipine and nifedipine) and verapamil to model and biological membranes by fluorescence analysis. The consistent location of Ca agonist Bay K 8644 was determined to be within the region of the first few methylene segments of the fatty acyl chains of the membranes (Mason et al. [180]). This position is near to that observed for the DHP calcium channel antagonists nimodipine and Bay P 8857.

The majority of studies on OS were performed with DHPs with various lipophilicity, but only a few studies reported amphiphilicity of DHP derivatives. Amphiphilic DHP derivative K-2-11 reduced the cellular generation of ROS. It also revealed complete reversal of multidrug resistance (MDR) of the resistant cells. K-2-11 was more efficient than well-known MDR inhibitor verapamil. Cytotoxic effects of anticancer drug doxorubicin were enhanced by K-2-11 in both MDR and parental, nonresistant cell line (Cindric et al. [181]). K-2-11 suppresses increase of ROS and consequentially prevents NF-*κ*B activation leading to decreased expression of MDR1 and increased expression of antiapoptotic genes. This signaling switch is necessary for restoring the chemosensitivity of cancer cells. This phenomenon is characteristic both for 1,4-DHPs [182] (18 novel asymmetrical DHPs bearing 3-pyridyl methyl carboxylate and alkyl carboxylate moieties at C3 and C5 positions, resp., as well as nitrophenyl or heteroaromatic rings at C4) and for their oxidized forms, pyridine compounds (Zhou et al. [183]).

### 3.4. Dependence of AOA of DHPs on the Experimental System

AO effect of DHPs depends on their structure and the experimental system used (*in vitro* model system, subcellular organelle, and cells,* ex vivo* and* in vivo*). Ideally, for the evaluation of the profile and value of DHPs AO properties, each compound should be tested in as many systems as possible (Dubur et al. [45]).

Lipidomics studies have been traditionally explored for studying AOA of DHPs. Proteomics methods are less represented and are mostly focused on the properties of DHPs related to scavenging of protein free radicals. So far, there are no studies on the role of DHPs in scavenging nitrosoperoxycarbonate, the reactive species formed out of peroxynitrite, in the presence of carbon dioxide. Although it was shown that albumin binds diludine, no studies revealed the relevance of this effect for the AOA of diludine.

There are findings showing that dihydropyridine calcium antagonists (DHPs CA) could indirectly play a beneficial, protective role during development of atherosclerosis. Namely, Berkels et al. [184] have studied antioxidative properties of four substances: the DHP prototype CA, nifedipine, the long-acting CA, lacidipine, the DHP calcium channel agonist, Bay K 8644, and the bulky DHP derivate, Bay O 5572, in three different models: (1) in an* in vitro* superoxide anion generating system (hypoxanthine/xanthine oxidase) for testing the “pure” antioxidative effect, (2) in an artificial membrane preparation (dimyristoylphosphatidylcholine) for mimicking a more physiological environment, and (3) under conditions of stimulated ROS release (hyperglycemia) from native endothelial cells derived from porcine coronary arteries.

The study also revealed the potential correlation between lipophilic and AO properties of DHPs. In the first model, Bay K 8644 was significantly more effective in scavenging superoxide anions than lacidipine, Bay O 5572, or nifedipine (micro- to millimolar concentration range). Addition of an artificial membrane preparation resulted in an enhanced AO effect, with lacidipine being the most effective DHP in quenching radicals (low micromolar concentration range). In the third model, mimicking hyperglycemia (30 mmol/L), nifedipine was significantly more potent antioxidant (therapeutical nanomolar concentration range) than the other DHPs. Calculated lipophilicity of these four substances (lacidipine > Bay O 5572 > Bay K 8644 > nifedipine) was positively correlated with antioxidative potential only in the second experimental model. It has been concluded that AO properties of DHP substances need to be tested in various models for demonstrating that nifedipine exhibits ROS-quenching properties in a therapeutic concentration range [184].

#### 3.4.1. AOA of DHPs in Isolated Cells and Cell Cultures (Comparison with Other Simplest Systems)

**Figure 4:**
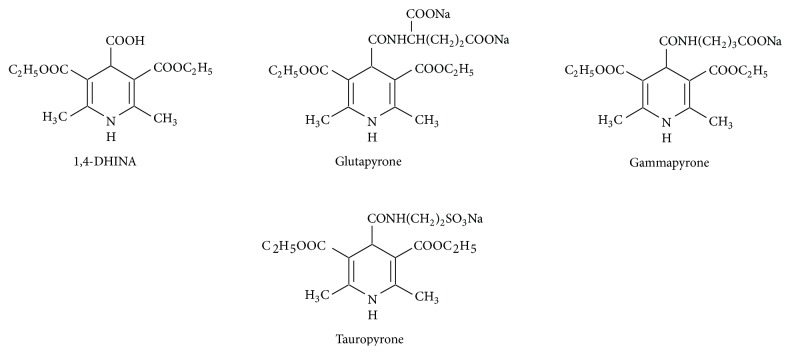
Derivatives of 1,4-dihydroisonicotinic acid (1,4-DHINA).

Although DHPs possess neuromodulatory and/or antimutagenic properties, the mechanisms of action related to these phenomena are not entirely elucidated. Borovic et al. [185] have studied 1,4-dihydroisonicotinic acid (1,4-DHINA) derivatives of 1,4-DHP, water-soluble analogues of a well-known AO diludine (diethone): 2,6-dimethyl-3,5-diethoxycarbonyl-1,4-dihydroisonicotinic acid, sodium 2-(2,6-dimethyl-3,5-diethoxycarbonyl-1,4-dihydropyridine-4-carboxamido)glutamate, glutapyrone and sodium 2-(2,6-dimethyl-3,5-diethoxycarbonyl-1,4-dihydropyridine-4-carboxamido)ethane-sulphate, tauropyrone as AO and bioprotectors (Figure 4).

1,4-DHINA's activities were studied in comparison to Trolox by N,N-diphenyl-N′-picrylhydrazyl (DPPH^•^), deoxyribose degradation, ABTS^•^ radical scavenging, and AOA (antioxidative capacity method) assays; copper-induced LP of cultured rat liver cells (MDA determination by HPLC and 4-hydroxynonenal-protein conjugates by dot-blot); ^3^H-thymidine incorporation and trypan blue assay for liver cells growth and viability. Ic decreased the amount of 4-HNE-protein adducts. In all assays, Ia was the most potent AO, able to completely abolish copper induced LP of liver cells, while Ic only slightly decreased it. Thus, AOA is important activity principle of Ia, which was even superior to Trolox in treated cell cultures. Ia (and its analogues) are easily oxidized in the Fenton system (Rubene et al. [186]), exerting ARA too.

### 3.5. Peculiarities Related to Antioxidative and Antiradical Activity of Some 1,4-DHPs: Ca Antagonists

Nine commercialized, structurally and functionally different DHPs, CA, will be discussed further. Their common feature is ability to prevent OS. This also counts for some of their metabolites, as already discussed. The comparative effects of some DHPs, CA, on oxidative stress-induced modification of LDL were already reviewed in Section 3.3.1 (2)-(a). AOA of CA DHPs was discussed in Sections 3.3.1 (2)-(b) and 3.3.1 (2)-(c).

#### 3.5.1. Nifedipine and Its Close Analogues

Nifedipine, verapamil, and antiarrhythmic-antihypoxic drug, stobadin, were shown to depress lipid peroxidation of phosphatidylcholine liposomes (Ondriaš et al. [187]). However, data obtained in some other experimental systems are conflicting.

In an* in vitro* model of sarcolemmal membrane lipid peroxidation, three calcium blockers (nifedipine, verapamil, and diltiazem) exhibited concentration-dependent (10–400 *μ*M) inhibitory effects [188, 189]. Nifedipine, the most effective calcium blocker, was more than two-fold potent compared to propranolol, achieving significant effect at 10 *μ*M. Nifedipine protective role on LP using reduced glutathione as model marker was recently described (Ray et al. [190]). Antiperoxidative properties of CA nifedipine and its analogues were explored in different systems/pathogenic processes: atherogenesis (Henry [165]), brain focal ischemia (Yamato et al. [191]), nephroprotection related to cyclosporine intake (Chander and Chopra [192]), and hepatoprotection related to intake of diethyldithiocarbamate (Gaafa et al. [193]). Recent data suggest that nifedipine action as protector for endothelial cells proceeds independently from its CA properties.

The absence of antioxidant effects of nifedipine and diltiazem on myocardial membrane lipid peroxidation, opposite to nisoldipine and propranolol, was also described [194]. Nisoldipine and propranolol were shown to have a concentration-dependent antiperoxidant effect, with IC_50_ values of 28.2 and 50.1 *μ*M, respectively. Finally, nisoldipine appeared to possess dual antiperoxidant mechanisms, involving both preventive and chain-breaking properties.

These findings were confirmed in some other studies, including reports on the lack of antioxidative activity of nifedipine and nicardipine, even at 500 *μ*M concentration in heart membrane lipid peroxidation tests [134]. Similarly, ROS formation in bovine aorta smooth muscle cells was not affected by addition of amlodipine, nimodipine, and nifedipine [123].

*(1) Metabolites of Nifedipine and Its Analogues as Antioxidants and Regulators of OS*. Antioxidant activity of nifedipine, 3,5-dimethoxycarbonyl-2,6-dimethyl-4-(2-nitrophenyl)-1,4-dihydropyridine, was originally studied* in vitro* by Kirule et al. [195] and Tirzit et al. [196]. According to the kinetic data of peroxide accumulation and the ESR spectra (inhibition of the autoxidation of methyl oleate in presence of nifedipine) AO action was exerted by the formation of nitroso analogue of the oxidized nifedipine, nitroso nifedipine: 2,6-dimethyl-4-(2-nitrosophenyl)-3,5-pyridine dicarboxylate (NO-NIF). This nitroso aromatic derivative can form nitroxyl radicals exhibiting remarkable AOA in the presence of unsaturated fatty acids and lipids [196].

The primary species of free radicals that have been obtained and identified were ion radicals of the nitrophenyl type (Ogle et al. [76]). Such a mechanism coincides with mechanisms proposed afterwards by Núñez-Vergara et al. [96], López-Alarcón et al. [103], Valenzuela et al. [104], Fukuhara et al. [174], and Yáñez et al. [197].

There are also data showing that nitroso compounds may inhibit LP by direct radical trapping and subsequent formation of stable nitroxide radicals. It was further found that the reactivity between the synthesized 1,4-DHP derivatives with alkylperoxyl radicals involves electron transfer reactions. This is documented by the presence of pyridine as a final product of the reaction and complete oxidation of the nitroso group in the case of the nitrosoaryl 1,4-dihydropyridine derivatives (Valenzuela et al. [104]). Tested compounds reacted faster toward alkylperoxyl radicals and ABTS radical cation than alkyl ones (López-Alarcón et al. [103]).

Nitrosonifedipine, a photodegradation product of nifedipine, significantly recovers cellular damage induced by tumor necrosis factor-alpha. It also prevents toxic effects of cumene peroxide which hampers integrity of cell membranes through oxidative stress. Its positive effects are equal to Trolox-C. As a result, nitrosonifedipine was already a long time ago claimed as a candidate for a new class of antioxidative drugs (Kirule et al. [195]), cellular protectors against oxidative stress in glomerular endothelial cells [174].

Moreover, Misik et al. [198], Ondriaš et al. [199], and Staško et al. [200] studied AOA of nifedipine and its oxidized nitroso analogue. NO-NIF prevents the progression of type 2 diabetic nephropathy associated with endothelial dysfunction through selective AO effects (Ishizawa et al. [201]). NO-NIF administration reduces albuminuria and proteinuria as well as glomerular expansion without affecting glucose metabolism or systolic blood pressure. NO-NIF also suppresses renal and systemic OS and decreases the expression of intercellular adhesion molecule-1 (ICAM-1), a marker of endothelial cell injury, in the glomeruli of the KKAy mice. Similar effects were achieved in endothelial nitric oxide synthase (eNOS) knockout mice. Moreover, NO-NIF suppresses urinary angiotensinogen (AGT) excretion and intrarenal AGT protein expression in proximal tubular cells in the KKAy mice. On the other hand, hyperglycemia-induced mitochondrial superoxide production was not attenuated by NO-NIF in cultured endothelial cells.

Fujii and Berliner found EPR evidence for free radical adducts of nifedipine* in vivo* [202]. The nature of these radicals was surmised by comparing the reaction of illuminated nitrosonifedipine with polyunsaturated fatty acids. Surprisingly, identical radical spectra were detected from excised liver doped with nonilluminated nifedipine, suggesting that this drug can be enzymatically converted* in vivo* to its nitroso analogue without the requirement for illumination. This is one of the first reports of* in vivo* EPR evidence for a class of unsaturated fatty acid radical conjugates resulting from the normal metabolism of a common drug.

Díaz-Araya et al. [173] studied some 4-nitrophenyl-DHPs on Fe^3+^ initiated LP in rat brain slices. LP, as measured by MDA formation, was inhibited by all the tested nitroaryl derivatives of 1,4-DHP over a wide range of concentrations. On the basis of both time course and IC_50_ experiments the tentative order of AOA on rat brain slices was nicardipine > nisoldipine > (R,S/S,R)-furnidipine > (R,R/S,S)-furnidipine > nitrendipine > nimodipine > nifedipine. 1,4-DHP derivatives that lack a nitro group in the molecule (isradipine and amlodipine) also inhibited LP in rat brain slices but at higher concentrations than that of nitro-substituted derivatives. All tested compounds reduced and oxidized nitrosoaryl derivatives (2,6-dimethyl-4-(2-nitrosophenyl)-3,5-pyridinedicarboxylic acid dimethyl ester (photooxidation product of nifedipine – NTP) a.o.) and were more potent inhibitors of LP than their parent molecules (Valenzuela et al. [104]).

The electrooxidation process of 4-nitrosoaromatic DHPs is a strongly pH-dependent (two-electron two-proton mechanism): ECEC type of mechanism, that is, the sequence:* e* ^−^/H^+^/*e* ^−^/H^+^ at pH > 8.5; ECCE mechanism (*e* ^−^/H^+^/H^+^/*e* ^−^) at pH < 8.5 dominates. Reduction reaction of nitroso group is as follows: R-NO + 2*e* ^−^ + 2H^+^ → RNHOH (Bollo et al. [203]).

#### 3.5.2. Lacidipine

It is a generic DHP type antihypertensive CA, 3,5-diethyl 4-{2-[(1*E*)-3-(*tert*-butoxy)-3-oxoprop-1-en-1-yl]phenyl}-2,6-dimethyl-1,4-dihydropyridine-3,5-dicarboxylate.

Ursini [204] described redox behaviour of lacidipine and showed its tissue protective features. Cristofori et al. studied antiatherosclerotic activity, in addition to lacidipine's CA and AO properties [205]. Lacidipine reduced the extent of atherosclerotic area in hypercholesterolemic apoE-deficient mice (these mice show widespread vascular lesions which closely resemble the inflammatory fibrous plaques seen in humans in atherosclerosis). The reduction may be associated with the capacity of the drug to maintain endothelial NO levels at concentrations useful to protect against vascular damage. This work suggested that DHPs modulate vascular relaxation* via* increased release of NO.

Herbette et al. [178] remarked optimal hydrophobicity of lacidipine due to cinnamic acid substituent, so membrane interactions and facilitation of the treatment of atherosclerosis could proceed (see also Section 3.3.1 (2)-(a)).

#### 3.5.3. Amlodipine

Amlodipine (*Norvasc*), (*RS*)-3-ethyl 5-methyl 2-[(2-aminoethoxy)methyl]-4-(2-chlorophenyl)-6-methyl-1,4-dihydropyridine-3,5-dicarboxylate (AML), has an antioxidant effect on vessels* in vitro* and is a 3rd generation of charged dihydropyridine CCB that is widely used for the treatment of hypertensive patients.

Amlodipine was shown to have the highest affinity (amlodipine > verapamil ≫ diltiazem) for the membrane bilayer (*K*_*p*_ = 10^4^). It produced the significant changes in membrane thermodynamic properties, including a reduction in the thermal phase transition temperature (−11%), enthalpy (−14%), and cooperative unit size (−59%), relative to the control, phosphatidylcholine liposomes (Mason et al. [49]).

Amlodipine AOA is related to its reductant nature or hydrogen donor properties, respectively. Its ability for donating protons and electrons to the lipid peroxide molecules blocks the LP process.

Amlodipine and even its enantiomers (Zhang et al. [206]) act as ROS and NOS effectors in several model systems of OS. Antioxidant properties of amlodipine were recently reviewed by Vitolina et al. [32]. Both* in vitro* and* in vivo* studies of amlodipine AO properties revealed inhibition of lipids oxidative damage, primarily those associated with cellular membranes and lipoprotein particles (LDL) (Mason et al. [50]).

Under controlled experimental conditions* in vitro* amlodipine showed AOA and ARA, by inhibition of lipid peroxide formation and trapping ROS. Its scavenging activity for hydroxyl and peroxyl radicals at concentrations as low as 10.0 nmol/L (which is remarkably less compared to the classical antioxidants, GSH, uric acid, and Trolox) was shown to be independent of the calcium channel modulation (Franzoni et al. [207]).

AML showed efficiency as scavenger of peroxyl radicals (TOSC assay: 5945 ± 544 units/mg), significantly stronger (>50%, *P* < 0.001) than GSH (2733 ± 636 units/mg) and 70% weaker (*P* < 0.0001) than uric acid (18144 ± 696 units/mg) and Trolox (17522 ± 734 units/mg).

Of interest, the scavenging capacity of AML towards hydroxyl radicals (1455 ± 154 units/mg) was 320% higher (*P* < 0.00001) than that of GSH (358 ± 112 units/mg), 20% higher than that of uric acid (1198 ± 121 units/mg), and 100% higher than that of Trolox (759 ± 143 units/mg).

Amlodipine was shown to increase enzyme activity of paraoxonase (PON) and glutathione peroxidase (GSH-Px). However, it also decreases glutathione reductase (GSSG-R) activity and diminishes the concentration of the endogenous antioxidant *α*-tocopherol (vitamin E). Moreover, AML in a concentration of 2 ng/mL decreased the content of malonic dialdehyde and activity of superoxide dismutase in the blood (Gatsura [208]).

Verapamil and amlodipine produced a potent anti-ischemic effect and reduced area of myocardial infarction in rats. The observed changes were accompanied by inhibition of LP. In contrast to verapamil,* in vitro* application of AML in a dose of 50 ng/mL decreased hemoglobin affinity for oxygen. When present in a concentration of 2 ng/mL, AMD decreased the content of MDA and activity of SOD in the blood.

On the other hand, amlodipine shows no activity related to inhibition of macrophage superoxide release and cell migration, which occurs as a consequence of decreased TNF*α* induced O_2_^•^ release.

Amlodipine-induced reduction of OS in the brain is associated with sympathoinhibitory effects in stroke-prone spontaneously hypertensive rats (SHRSP) (Hirooka et al. [209]). Antihypertensive treatment with amlodipine reduced OS in all examined areas of the brain and decreased blood pressure without a reflex increase in sympathetic nerve activity. Nicardipine, another CA DHP, surprisingly, was significantly less active than amlodipine.

#### 3.5.4. Lercanidipine

Tomlinson and Benzie reported AO effect of lercanidipine [210], which is well known as antihypertensive drug Zanidip, 2[(3,3-diphenylpropyl)(methyl)-amino]-1,1-dimethylethyl methyl 2,6-dimethyl-4-(3-nitrophenyl)-1,4-dihydropyridine-3,5-dicarboxylate. Comparative data about this drug AOA were presented in parts of this paper about other individual CA DHPs and in the part about the* ex vivo* DHPs effects on LDL.

#### 3.5.5. Nimodipine

Nimodipine (ND), commercially known as Nimotop, is 3-(2-methoxyethyl) 5-propan-2-yl 2,6-dimethyl-4-(3-nitrophenyl)-1,4-dihydropyridine-3,5-dicarboxylate. It is centrally active CA.

Treatment with glutathione blocked and with nimodipine attenuated neuronal cell death, caused by prolonged exposure of cell culture to 4-HNE (Faraqui [211]).

Nascimento et al. [212] found AO effect of nimodipine in young rats after pilocarpine- (PIL-) induced (in 400 mg/kg) seizures. The PIL administration increased the striatal catalase (CAT) activity. The administration of ND, 30 mg/kg, 30 min before PIL, preserved normal value of CAT activity. On the other hand, no difference was detected in the animals treated with lower dose, 10 mg/kg. These results confirm the neuroprotective/antiepileptic effect of ND in young rats, suggesting that this drug acts positively on lipid peroxidation (in both doses). Nimodipine cannot induce these effects* via* blockade of Ca^2+^ channel.

Ismailoglu et al. [213] studied the therapeutic effects of melatonin and nimodipine in rats after cerebral cortical injury. These beneficial effects in rats after cerebral cortical injury seemed to be related to AOA of nimodipine.

#### 3.5.6. Benidipine

Licensed in Japan and South Asia as CA (CCB) benidipine possesses AO properties. Chemically, it is 5-methyl 3-[(3*R*)-1-(phenylmethyl)piperidin-3-yl] 2,6-dimethyl-4-(3-nitrophenyl)-1,4-dihydropyridine-3,5-dicarboxylate (or its hydrochloride, (4R)-rel-3,5-pyridinedicarboxylic acid, 1,4-dihydro-2,6-dimethyl-4-(3-nitrophenyl)-, 3-methyl 5-[(3R)-1-(phenylmethyl)-3-piperidinyl] ester, hydrochloride (1 : 1)).

Benidipine influences processes connected with OS in several ways. It prevents lysophosphatidylcholine- (lysoPC)- induced injury and ROS production in human aortic endothelial cells (HAECs) (Matsubara and Hasegawa [214]). Matsubara et al. [215] explained this effect, based on stimulation of nitric oxide release.

LysoPC is a component of oxidized low-density lipoproteins (oxLDLs), which plays an important role in the pathogenesis of atherosclerosis. Pretreatment with benidipine (0.3–3 *μ*mol/L) for 24 h protected against lysoPC-induced cytotoxicity in the HAECs through inhibition of both lysoPC-stimulated ROS production and caspase-3/7-like activation, with a similar potency. Since caspase-3/7 is involved in executing the apoptotic process, the reduction of the activity of this enzyme by benidipine may explain the antiapoptotic effect of the drug. However, benidipine did not suppress lysoPC-induced phosphorylation of mitogen-activated protein kinases and Ca^2+^ influx in HAECs. These results suggest that the antioxidant properties of benidipine may be responsible for its ability to inhibit ROS production, a possible reason for reduced activation of caspase-3/7. In conclusion, benidipine suppresses lysoPC-induced endothelial dysfunction through inhibition of ROS production, which is due at least in part to its antioxidant effect, and not through the inhibition of L-type voltage-dependent calcium channels.

Matsubara and Hasegawa [216] examined the effects of benidipine on cytokine-induced expression of adhesion molecules and chemokines (chemoattractants), which are important for the adhesion of monocytes to endothelium. Pretreatment of HAECs with benidipine (0.3–10 *μ*mol/L) for 24 h significantly suppressed cytokine-induced vascular cell adhesion molecule-1 (VCAM-1) and intracellular cell adhesion molecule-1 (ICAM-1) mRNA and protein expression, resulting in reduced adhesion of THP-1 monocytes. Benidipine also suppressed induction of monocyte chemoattractant protein-1 (MCP-1) and interleukin-8. Benidipine also inhibited redox-sensitive transcriptional nuclear factor-*κ*B (NF-*κ*B) pathway, as determined by Western blotting of inhibitory *κ*B (I*κ*B) phosphorylation and luciferase reporter assay. Results of analysis using optical isomers of benidipine and antioxidants suggest that these inhibitory effects were dependent on pharmacological effects other than Ca^2+^ antagonism. Benidipine may thus have anti-inflammatory properties and benefits for the treatment of atherosclerosis.

Benidipine was also shown to inhibit ROS production in polymorphonuclear leukocytes and oxidative stress in salt-loaded stroke-prone spontaneously hypertensive rats (Matsubara et al. [217]).

It should be mentioned that other DHPs also have endothelial AO actions [218].

#### 3.5.7. Azelnidipine (AZL)

Azelnidipine, 3-[1-[di(phenyl)methyl]azetidin-3-yl] 5-propan-2-yl 2-amino-6-methyl-4-(3-nitrophenyl)-1,4-dihydropyridine-3,5-dicarboxylate (AZL), CAS number: 123524-52-7, is commercially available 4-nitroaryl-DHP type calcium antagonist with long-acting antihypertensive action (long-acting CA (CCB)) and a low reported incidence of tachycardia. It additionally possesses beneficial effects in OS and diabetic conditions.

Azelnidipine prevents cardiac dysfunction in streptozotocin-diabetic rats by reducing intracellular calcium accumulation (altering intracellular Ca^2+^ handling proteins), OS, and apoptosis (Kain et al. [219]). AZL can reduce the superoxide production. It exerts its protective effects by targeting the NADPH oxidase and mitochondrial redox enzymes. AZL-treated diabetic rats express enhanced level of bcl-2 in the lysates of heart muscle indicating that AZL plays protective role in cardiac apoptosis.

It has been previously observed that azelnidipine inhibits tumor necrosis factor-alpha-induced endothelial cell (EC) oxidative stress through its AO properties (Nakamura et al. [220]). Azelnidipine, but not nitrendipine, completely inhibits the Ang II-induced ROS generation in ECs (Matsui et al. [221]).

Furthermore, azelnidipine, but not nitrendipine, was found to partially restore decreased pigment epithelium-derived factor (PEDF) mRNA levels in Ang II-exposed ECs. This study suggests that AZL influence depends on its antioxidative properties. Authors concluded that upregulation of PEDF by azelnidipine may become a therapeutic target for the treatment of diabetic retinopathy associated with hypertension.

Antihypertensive agents with AO effects are potentially useful for diabetic patients with hypertension. While DHP type CA are among the most frequently used antihypertensive drugs, azelnidipine has been reported to have a unique AO effect* in vitro* and* in vivo*, in experimental animals (Ohmura et al. [222]). In hypertensive diabetic patients, azelnidipine treatment for 12 weeks induced a more significant decrease in erythrocyte LOOH level than amlodipine, although the values related to blood pressure during each treatment remained comparable. These data confirm the usefulness of LOOH level in erythrocyte membrane as a marker of OS* in vivo* and indicate that azelnidipine has a unique antioxidative property in humans.

Daikuhara et al. [223] reported the results of the OLCA study, based on combination of (1) olmesartan and a calcium channel blocker (azelnidipine) or (2) candesartan and a CCB amlodipine in two groups of diabetic hypertensive patients. Patients treated with the first combination presented highly persistent early morning antihypertensive effect and stronger decrease in heart rate, fasting blood glucose and HbA1c levels, and microalbuminuria, when compared to patients treated with the combination (2). Because diabetes is associated with severe chronic OS the observed results might be at least in a part due to the AOA of azelnidipine.

In favor of this are findings of Abe et al. [224] who found additive antioxidative effects of azelnidipine on angiotensin receptor blocker olmesartan treatment for type 2 diabetic patients with albuminuria.

Similarly, the AOA of thiazolidinediones (insulin sensitizer) and their effect on cardiovascular function in type 2 diabetic model rats and also those of some DHPs (nifedipine, amlodipine, or AZL, commonly used antianginal and antihypertensive agents) in cultured human endothelial cells LP were examined (Mizushige [225]). The AOA was evaluated by measuring 8-iso-prostaglandine F_2*α*_ concentration and azelnidipine exhibited potent AOA.

Insulin (INS) resistance combined with hyperinsulinemia is involved in the generation of OS. A relationship exists between increased production of ROS and the diverse pathogenic mechanisms involved in diabetic vascular complications, including nephropathy. Manabe et al. [226] revealed that high doses of INS augmented mesangial cell proliferation through generation of intracellular ROS and activation of redox signaling pathways. Cell proliferation was increased in a dose-dependent manner by high doses of INS (0.1–10 *μ*M) but was inhibited by 0.1 *μ*M AZL. Namely, the INS-increased phosphorylation of mitogen activated protein kinase/extracellular signal-regulated kinase 1/2 (MAPK/ERK 1/2) was inhibited by 0.1 *μ*M AZL. The same AZL concentration blocked intracellular ROS production more effectively than 0.1 *μ*M nifedipine. The NADPH oxidase inhibitor, apocynin (0.01–0.1 *μ*M), prevented INS-induced mesangial cell proliferation. So, azelnidipine inhibits insulin-induced mesangial cell proliferation by inhibiting the production of ROS. Therefore azelnidipine may have the potential to protect against the onset of diabetic nephropathy and slow its progression.

Azelnidipine inhibited H_2_O_2_-induced cell death in neonatal rat cardiomyocytes (Koyama et al. [227]). Azelnidipine and nifedipine did not affect the H_2_O_2_-induced activation of extracellular signal-regulated protein kinases (ERK) and p38 MAPK (mitogen-activated protein kinase). In contrast, azelnidipine, but not nifedipine, inhibited H_2_O_2_-induced c-Jun NH_2_-terminal kinases (JNK) activation. Authors concluded that azelnidipine has inhibited the H_2_O_2_-induced JNK activation and cardiac cell death. Therefore azelnidipine may have cardioprotective effects against OS.

A specific atheroprotection activity of azelnidipine relates to inhibition of TNF-*α*-induced activator protein-1 activation and interleukin-8 expression in human umbilical vein endothelial cells (HUVEC), through suppression of NADPH oxidase-mediated reactive oxygen species generation (Nakamura et al. [220]). TNF-*α* could play a central role in pathogenesis of insulin resistance and accelerated atherosclerosis in the metabolic syndrome. The concentration of AZL found to be effective in these* in vitro* experiments is within therapeutic range. As EC do not possess voltage-operated L-type calcium channels, it is suggested that the beneficial effects of azelnidipine are not likely due to CA property but to its unique AO ability. Furthermore, it has been recently found that serum levels of monocyte chemoattractant protein-1, a biomarker for subclinical atherosclerosis, were significantly decreased by the AZL treatment in patients with essential hypertension. In this paper [220], authors hypothesize that, due to its unique TNF-*α* signal modulatory activity and antioxidative property, azelnidipine may be a promising DHP for targeting diabetes and cardiovascular diseases in hypertensive patients with metabolic syndrome.

Shinomiya et al. [228] evaluated its AOA in cultured human arterial EC, under OS. Azelnidipine has shown a potent antioxidative effect that could be of significant clinical benefit when combined with its long-lasting antihypertensive action and low incidence of tachycardia.

Azelnidipine inhibited TGF-*β*1 and angiotensin II- (Ang II-) activated *α*1(I) collagen mRNA expression in hepatic stellate cells (HSCs) (Ohyama et al. [229]). Furthermore, TGF-*β*1- and Ang II-induced OS and TGF-*β*1-induced p38 and JNK phosphorylation were reduced in HSCs treated with AZL. Azelnidipine significantly decreased inflammatory cell infiltration, profibrotic genes expression, HSC activation, LP, oxidative DNA damage, and fibrosis in liver of CCl_4_- or TAA-treated mice. Finally, AZL prevented decrease of the expression of some AO enzymes and accelerated regression of liver fibrosis in CCl_4_-treated mice. Hence, the antifibrotic mechanism of AZL against CCl_4_-induced liver fibrosis in mice may have been due to an increased level of AO defense. As azelnidipine is widely used in clinical practice without serious adverse effects, it may provide an effective new strategy for antifibrotic therapy.

#### 3.5.8. Manidipine

Manidipine, (2-[4-(diphenylmethyl)piperazin-1-yl]ethyl methyl 2,6-dimethyl-4-(3-nitrophenyl)-1,4-dihydropyridine-3,5-dicarboxylate), is a DHP CCB with reported nephroprotective properties. Calò et al. [230] studied effect of manidipine on gene expression and protein level of OS related proteins: p22(phox) (human neutrophil cytochrome b light chain (CYBA)) and heme oxygenase-1, HO-1. Relevance for antihypertensive effects was revealed. The study assessed the effect of manidipine on normal subjects' monocyte gene and protein expression of OS related proteins such as p22phox, a NADPH oxidase system subunit, critical in generating O_2_^•−^, and HO-1, induced by and protective against OS. Manidipine was compared with the ACE inhibitor captopril and the CCB nifedipine, in the presence and in the absence of sodium arsenite (NaAsO_2_) as an inducer of OS. Monocyte p22phox (CYBA) mRNA production was reduced by both manidipine and captopril, while no changes were induced by nifedipine. Manidipine increased monocyte HO-1 mRNA production, while nifedipine and captopril showed no effect. The effects of manidipine on p22phox and HO-1 gene expression in the presence of OS were also confirmed at the protein level. Thus, manidipine seems to suppress p22phox and to increase the HO-1 mRNA production and protein level. The manidipine-induced increase of HO-1 gene and protein expression seems to be a peculiar effect of this drug since it is not observed with captopril and nifedipine. This effect, together with the reduction of p22phox mRNA production, could play a role in its protective mechanism against OS.

#### 3.5.9. Mebudipine

The protective effect of mebudipine (1,4-dihydro-2,6-dimethyl-4-(3-nitrophenyl)-3,5-pyridinedicarboxylic acid 3-methyl-5-tert-butyl ester; BAY-n-6391) was revealed on OS and LP (MDA decrease, SOD, GPX, and catalase increase) in myocardial ischemic-reperfusion injury in male rats (Ghyasi et al. [231]).

There are articles about other commercial and experimental DHPs on OS, but we have reviewed only the most commonly studied compounds. Effects of other commercial CA DHPs on OS are also mentioned in several parts of this review.

### 3.6. 1,4-DHPs: Ca Agonists and Their AOA and ARA

For the most popular calcium agonist DHP Bay K 8644 no reaction with peroxyl radicals was registered (Toniolo et al. [114]). However, interaction with other compounds possessing AOA and ARA (quercetin) was found.

Opposite to that, AO N-acetylcysteine (NAC) diminished increase in Ca^2+^ transient amplitude and cell shortening induced by ISO and forskolin, whereas NAC had no effect on the (S)-(−)-methyl-1,4-dihydro-2,6-dimethyl-3-nitro-4-(2-trifluoromethylphenyl)pyridine-5-carboxylate—(−)-Bay K 8644-induced increases (Andersson et al. [232]).

Increased vasoconstriction responses to Bay K 8644 (3 × 10^−7^–3 × 10^−5 ^M) were significantly decreased by pyridoindole antioxidant stobadine treatment in diabetes (Ceylan-Isik et al. [233]).

The functional interaction between two L-type Ca^2+^ channel activators, quercetin and Bay K 8644, has been investigated in vascular smooth muscle cells. Biological ARA compound quercetin at nutritionally meaningful concentrations limited the responsiveness of vascular L-type Ca^2+^ channels to the pharmacological stimulation operated by Bay K 8644. These data contribute to a better understanding of quercetin effects on experimental* in vivo* cardioprotection (Saponara et al. [234]). Thus, these findings indicated that although Bay K 8644 does not exert potent and direct AOA, yet acting as calcium agonist it may affect efficiency of AO substances and* vice versa*.

Interaction of grapefruit juice (containing quercetin and its analogues) with DHPs CA diminished effectiveness of CA action of DHPs (Sica [235]).

### 3.7. Interpretations of the Mechanism(s) of Radical Scavenging and AOA by DHPs

#### 3.7.1. Molecular Mechanism(s) of Radical Scavenging and AOA of DHPs in the Model Systems

3,5-Dicarbonyl-1,4-dihydropyridine derivatives possess powerful bis-*β*-carbonylvinyl-amino conjugation and for that reason cannot be considered as ordinary amino antioxidants. The electron and/or H donation from DHPs ensures their reductant role and results in AOA and ARA. Oxidation reaction from DHPs results in production of corresponding heteroaromatic pyridine derivatives.

Detailed studies were made about substituent in DHP ring positions: 1,4-, namely, 4-unsubstituted-; 4-substituted: 4-alkyl-; 4-aryl-; 4-alkylaryl- a.o.; 2,6-; 3,5- (diacetyl or dialkoxycarbonyl chain a.o.) electronic and steric effects on AOA and ARA of DHPs [44, 45, 51], see Sections 3.3.1 and 3.5. The bell-shaped dependence of DHPs AOA on the 3,5-dialkoxycarbonyl- chain length was observed [44, 45, 107, 109, 112], with the maximum activity at C_2_H_5_–C_4_H_9_. Decrease of AOA and incorporation into liposomes for DHPs with alkyl chains longer than R > C_4_H_9_ further were clarified as probable tendency to self-aggregation of these compounds ([51] and citation number 245 therein). Electron acceptor/electron donor properties are relevant for expression of AOA or ARA of 3,5-disubstituted DHPs. 3,5-Diacyl- substituted and 3,5-dicarbanilido- and 3,5-dipyridylamido-substituted DHPs are more active as AOs as their 3,5-dicyano-substituted analogues, which have electron acceptor properties [186].

Dubur et al. [89] observed overwhelming steric influence of substituents in position 4 of the DHP ring. Gaviraghi et al. [163, 164] proposed that AO activity of DHPs is partly dependent on capacity of the 1,4-DHP ring to donate electrons to the propagating radical (ROO^•^ or RO^•^) and to reduce it to a less reactive form. The abstraction (donation) of electron and/or H in the oxidation and LP reactions takes place from all 3,5-dicarbonyl-1,4-DHP systems and results in the formation of corresponding pyridine derivatives (Augustyniak et al. [51]). The physicochemical mechanism of ARA and AOA of 1,4-DHP has been extensively studied and discussed (Mulder et al. [236]), but precise mechanisms of their activity need further clarification.

The reactivity of C-4 substituted 1,4-DHPs possessing either secondary or tertiary nitrogen atom in the DHP ring toward alkyl, alkylperoxyl radicals, and ABTS radical cation was determined in aqueous media at pH 7.4 [103]. These compounds reacted faster toward alkylperoxyl radicals and ABTS radical cation than alkyl ones. N-Ethyl-substituted DHPs showed the lowest reactivity.

The 4-methyl substituted DHP was the most reactive compound in previously mentioned reactions (López-Alarcón et al. [103]). However, it was less active (0.68 versus 1.0) than Trolox-C. DHPs having electron-donating substituents (4-Me-DHP and* p*-MeO-Phe-DHP) showed the highest kinetic rate constants toward ABTS radical cation;* p*-nitro-Phe-DHP, a compound with an electron-withdrawing substituent, showed a lower kinetic rate constant; and N-alkyl-DHP compounds show kinetic rate constants lower than the -NH-DHP.

Hydrogen at the 1-position of the DHP ring was revealed, according to the deuterium kinetic isotope effect studies, to be involved in the proposed ARA mechanism. This fact is mostly noticeable in the case of alkyl radicals. N-Ethyl-substituted DHPs show the lowest reactivity when compared to Trolox or nisoldipine. In all cases, the respective pyridine derivative was detected as the main product of the reaction (López-Alarcón et al. [103]). Authors indicate that the kinetic rate constants toward alkyl, alkylperoxyl, and ABTS radical cation depend on the nature of the substituents in the C-4 position of DHP and the presence of the secondary amine group in the dihydropyridine ring, that is, the presence of the hydrogen in 1-position.

Yáñez et al. [197] have studied the reactivity of 11 derivatives of 1,4-DHPs (including commercial CA) with alkylperoxyl radicals and ABTS radical cation. The tested 1,4-DHPs were 8.3-fold more reactive towards alkylperoxyl radicals than to the ABTS cation radical. All commercial 1,4-DHP type CCBs were weaker than Trolox-C. The participation of the hydrogen atom in the 1-position appears to be very relevant for exhibited reactivity. Hantzsch ester (diludine) was again found to be the most active compound in the reaction with alkylperoxyl radicals, 2.3-fold more active than Trolox. The photodegradation product of nifedipine (nitrosophenyl derivative of pyridine) also showed a high activity. Kinetic rate constants for the reaction between 1,4-DHP compounds and alkylperoxyl radicals exhibited a fairly good linear correlation with the oxidation peak potential of DHP derivatives. However, the activity of tested 1,4-DHPs towards ABTS radical cation showed an independence between kinetic rate constants and oxidation peak potentials.

Kirule et al. [195] and Tirzit et al. [196] studied mechanism of AOA of 4-nitrophenyl-1,4-DHPs, nifedipine and its analogues, involving formation of 4-(2′-nitrosophenyl)-pyridine derivative (as active principle) as a result of intramolecular redox reaction, using chemical, electrochemical, and biochemical approaches (see Sections 3.3.1 and 3.5.1).

Núñez-Vergara et al. [237] reported the electrochemical oxidation of C4-hydroxyphenyl-substituted 1,4-DHP derivatives. The oxidation proceeds* via* formation of the unstable dihydropyridyl radical, as confirmed by controlled-potential electrolysis (CPE) and ESR experiments. This type of 1,4-DHPs has significant activity towards the radicals even when compared with commercial 1,4-DHP drugs with well-known antioxidant ability.

It was observed that nicardipine preferentially targets RO^•^ radicals and is inactive against ROO^•^. Lacidipine, on the other hand, is equally active towards both types of radicals (Gaviraghi et al. [164]). The cytoprotective effect against exposure to H_2_O_2_ was more significant for lacidipine (ID_50_ = 14 nM, its log⁡*P* = 5.4, membrane partition = 136000, assumes position located 7 Å near to the membrane center; other less lipophilic DHPs located 12–16 Å far from the center) as compared to amlodipine, nifedipine, and nicardipine, in smooth muscle cell culture (Gaviraghi et al. [164]). Oxidative effect of H_2_O_2_ shifts the Ca channel toward an open state. Thus, the redox property of CCBs DHPs may augment their CCB properties.

Oxidation of pharmacologically active Hantzsch 1,4-dihydropyridines was found by electrogenerated superoxide, using a voltammetric approach in DMSO solutions (Ortiz et al. [238] and Ortiz et al. [239]). Raghuvanshi and Singh [240] have also reported oxidative aromatization of these DHPs, induced by superoxide.

Chemiluminescence (CL) was used in the studies analyzing the antioxidant activity of 12 various 4-flavonil-1,4-dihydropyridine derivatives (Kruk et al. [241]) on a chemical system involving a superoxide radical anion. These derivatives showed structural similarity to flavonoids, with respect to the presence of rings A, B, and C. The results obtained in this study indicate that the tested derivatives may catalyze conversion of superoxide radicals, through mimicking the activity of superoxide dismutase by delivering H^+^ for reaction:

(3) O2−•+O2−•+2H+⟶H2O2+O12

The enhanced emission of the light in the presence of tested compounds was significant and related to stimulated production of H_2_O_2_ and ^1^O_2_ from O_2_^•^. The latter species were removed from the reaction mixture by the following sequence of reactions:

(4) H+O2−•⟶HO2•HO2•+O2•−⟶HO2−+O122HO2•⟶H2O2+O122O12⟶O22+hν∗

or to take part in spontaneous dismutation of H_2_O_2_:

(5) 2H2O2⟶2H2O+O12

The authors have offered an original concept of action for 4-flavonil-1,4-dihydropyridine derivatives unrelated to that of O_2_^•^ radical-trapping, chain-breaking antioxidants. Instead, they showed that these compounds act similar to superoxide dismutases, converting O_2_^•^ to H_2_O_2_. Hydrogen peroxide is less toxic for cells than O_2_^•^ because it is predominantly removed by peroxidases and catalases. Finally, AO effect of these DHPs differed from those mediated by flavonoids with a catechol structure of ring B, which are well-known ^1^O_2_ quenchers.

Mulder and collaborators came to similar conclusions, especially related to molecular mechanisms of antioxidative activity of DHPs [236]. The AO properties of Hantzsch 1,4-dihydropyridine esters and two dibenzo-1,4-dihydropyridines, 9,10-dihydroacridine (DHAC) and N-methyl-9,10-dihydroacridine (N-Me-DHAC), have been explored by determining the autoxidation of styrene or cumene at 30°C. These experiments showed that Hantzsch esters are virtually inactive as chain-breaking antioxidants (CB-AOs), contrary to the findings observed by López-Alarcón et al. [103] who used CB-AOA in aqueous media at pH 7.4. Their reactivity toward peroxyl radicals was shown to be some 5 orders of magnitude lower than that of the excellent CB-AO, 2,2,5,7,8-pentamethyl-6-hydroxy-chroman (PMHC).

DHAC was found to be ~10 times less reactive than PMHC kinetic measurements using DHAC, N-deuterio-DHAC, and N-Me-DHAC, pointing out the abstraction of N-H hydrogen in DHAC by peroxyl radicals, despite the fact that the calculated C-H bond dissociation enthalpy (BDE) in DHAC is about 11 kcal/mol lower than the N-H BDE. The rates of hydrogen atom abstraction by the 2,2-diphenyl-1-picrylhydrazyl radical (DPPH^•^) have also been determined for the same series of compounds. The trends in the peroxyl^•^ and DPPH^•^ rate constants were found to be similar [236].

Tirzit et al. [242] have observed quenching of singlet oxygen by DHPs. This observation paved the ground for further research related to reactions of DHPs with hydroxyl radicals (Tirzit et al. [243]), singlet oxygen (Kazush et al. [94]), and mechanisms of action. A series of 1,4-DHP derivatives in NAD-H-Cu^2+^-H_2_O_2_ system inhibited forming of the hydroxyl radical (HO^•^), while 1,4-DHP derivatives with electron donor substituents in the molecule were shown to be capable themselves of generating HO^•^ in the presence of Cu^2+^ and H_2_O_2_. Rubene et al. [186] also described interaction of 1,4-DHP derivatives with Fenton's reagent, which produces hydroxyl radical (HO^•^). Rate constants of the DHPs reaction (1st order) with HO^•^ radical were high: in the range 10^9^ L × mol × sec^−1^, close to that of NADH, cysteine and thiourea. 3,5-Diacetyl- derivatives reacted faster compared to 3,5-dialkoxycarbonyl- ones. The reaction rate decrease was observed in the case of substitution at position 4 as compared to 4-unsubstituted DHPs. Some DHPs having electron donor -COO^−^ groups in the 3,5- or 2,6- positions of DHP ring reacted immediately (having rate constants higher as 10^9^ L × mol × sec^−1^). Rate constants with HO_2_^•^ and O_2_^•−^ radicals were of lower degree. Thus DHPs acting as oxygen radical scavengers could effectively inhibit ROS related reactions of LP initiation stage.

Nifedipine and nitrendipine reactivity toward singlet oxygen was also studied [244]. Nifedipine was shown to be a good scavenger of excited oxygen, mainly* via* physical deactivation with values of the total rate constant ranging from 20.8 × 10^5^ M^−1^ s^−1^ (in dioxane) to 93.0 × 10^5^ M^−1^ s^−1^ (in propylene carbonate). The less reactive pathway generated a photooxidation product. For that reason, a mechanism involving a perepoxide-like encounter complex in the first step of the reaction path was proposed (see [244], Figures 8 and 9 therein). The dependence was observed on solvent microscopic parameters of the total rate constant for the reaction between singlet oxygen and 1,4-DHPs. These findings show that nifedipine possesses stronger protective activity in biological systems than nitrendipine.

Density-functional-theory (DFT) calculations made by Wang et al. [245] confirmed the former experimental observations that Hantzsch ester, diludine, is potent antioxidant with high H atom-donating ability and relatively low prooxidant activity. Possible reaction pathways for radicals derived from 1,4-dihydropyridine and the resonance modes for radical species were given [245].

Moreover, two ethoxycarbonyl (EtOCO) substituents at C(2) and C(6) should further enhance Hantzsch ester, diludine H-atom-donating ability due to resonance effects. However, DHPs should be used in nonpolar rather than in polar solvents since, in the latter, not H-atom but electron transfer is preferred in the radical scavenging process [245].

Mulder et al. [246] proposed that quantum-thermochemical calculations must be used with caution to indicate “a promising lead antioxidant,” as they criticized the density-functional-theory (DFT) calculations made by Wang et al. [245].

#### 3.7.2. Possible Mechanisms of DHPs ARA and AOA in the Biological Systems: Interaction with Other OS Modifiers

Some of these mechanisms were already described (Sections 3.3.1 (2)-(b); 3.3.1 (2)-(c); 3.3.1 (3)-(b); 3.5).

Enzymatic sources of ROS with confirmed functional role in hypertension are NADPH oxidase, NO synthase (NOS), xanthine oxidase, and cyclooxygenase. Godfraind [3] has reviewed AO effects and protective action of calcium channel blockers (CCBs). Yao et al. [247] observed antioxidant effects (as inhibition of LP) for cardio- and neuroprotective CCBs (3–300 *μ*mol/L), including 7 DHPs, in homogenates of rat brain. IC_50_ values (*μ*M) were as follows: nifedipine (51.5) > barnidipine (58.6) > benidipine (71.2) > nicardipine (129.3) > amlodipine (135.5) > nilvadipine (167.3) > nitrendipine (252.1) > diltiazem (>300) = verapamil (>300). There are also research articles describing the AO properties of CCBs through direct scavenging effect or through preservation of the endogenous SOD activity. These findings indicate that CCBs may also act by reducing the production of vasoconstrictors, angiotensin, and endothelin.

When present in concentrations that can be achieved in plasma, CCBs may inhibit LP formation [3]. This AO activity seems to be typical for high lipophilic CCBs because their chemical structure facilitates proton-donating and resonance-stabilization mechanisms that quench the free radical reaction. Their insertion in the membrane, near polyunsaturated fatty acids at relatively high concentrations, potentiates proton donation (or atomary H) to lipid peroxide molecules, thereby blocking the peroxidation process. The remaining unpaired free electron associated with the CCB molecule can be stabilized in well-defined resonance structures associated with the DHP ring (Mason et al. [48]).

The radical reaction (according to Godfraind [3]) that describes the AO effects of a DHP CCBs is LOO^*∗*^ + DHP → LOOH + DHP^*∗*^ (where LOO^*∗*^ is lipid peroxide radical), which in general is reaction (7) of the LP reaction cascade consisting of ~10 reactions (Scheme 2) [89].

As the rate constants of* in vitro* interaction of 4-substituted DHPs with peroxyl radicals are three orders of magnitude lower than that of the vitamin E derivative, these DHPs must be considered as weak AO (Ursini [204]). However, due to partition coefficient of DHPs in membranes and in case of specific binding, high local concentration of DHPs may be obtained.

DHPs without CCB properties, for instance Bay w 9798, although structurally related to nifedipine, inhibit TNF-*α*-induced vascular cell adhesion molecule-1 expression in endothelial cells by suppressing reactive oxygen species generation [248].

Mitrega et al. [249] have discovered that antiarrhythmic and hemodynamic effects of oxidized heteroaromatic DHPs, oxy nifedipine, oxy nimodipine, oxy nitrendipine, and oxy nisoldipine, suggest that CCB DHPs and their metabolites could act at least in two ways: targeting OS related events as reductants (see Section 3.5.1 (1)) and/or bypassing OS related metabolic routes. Authors postulated that, contrary to current belief, NIF metabolites are pharmacologically active. ATP sensitive potassium channels were mentioned as a target.

### 3.8. DHPs: Anti- or Prooxidants?

Several substances (ascorbic acid being the most commonly studied) can serve either as antioxidants or as prooxidants, depending on given conditions (Herbert [250]). Therefore, Halliwell [251] has reported dilemma related to polyphenols as possible antioxidants and prooxidants, causing experimental artifacts (about 25) by oxidation of antioxidant compounds in the cell culture media. Nevertheless, it is generally accepted opinion that polyphenols act as antioxidants* in vivo*. Studies on DHPs also face such a dilemma. The exact roles (anti- or prooxidative) of any specific DHP depend on its structure, applied/achieved concentration, and specificity of the target/experimental testing system.

This situation resembles the case of antioxidative effects of tocopherol, which depends on the fate of the secondary radical as proposed by Winterbourn [252]. The question was “Vitamin E - Pro- or Antioxidant?”:

Antioxidant:

(6) LOO•+Toc⟶LOOH+Toc•Toc•=α-tocopheryl  radicalToc•+LOO•⟶chain  termination  of  lipid  peroxidation

Prooxidant:

(7) $$\begin{array}{c} {Toc}^{•}+\text{Lipid-H}⟶{Lipid}^{•}\left(\;\text{in LDL particles}\;\right) \end{array}$$

This example shows that, generally speaking, any AO must fulfil several criteria to be considered as an effective compound physiologically:

- It must be able to functionally interact with endogenous scavengers, even at low concentrations.
- It must affect endogenous pathways of OS.
- It should not have undesirable adverse effect.
- It should manifest the antioxidant efficacy dependent on the oxidant.
- It must discriminate among different strategies needed for 1-electron and 2-electron processes.
- Radical scavengers can be prooxidant unless linked to a radical sink (Winterbourn [252]).

According to these statements, DHPs could be effective as AO under physiological conditions* in vivo* (Godfraind [3] and others [30, 31, 38]) and* in vitro* in various experimental systems (cell and tissue) (see Sections 3.3; 3.4; 3.5).

Hence, calcium antagonists appeared to disrupt the fine balance between the production and scavenging of ROS. Nifedipine, verapamil, and diltiazem were shown to induce significant oxidative stress in the epididymal sperm (increased MDA and decreased catalase and superoxide dismutase activity). This may be the reason for the induction of male infertility [253].

The dualism of 1,4-DHP effects has been recorded as inhibition or promotion of LP, as quenching or initiating oxygen and nitrogen free radical chain reactions, radioprotecting or radiosensitizing, antagonizing or synergizing Ca^2+^ in electromechanical coupling, as well as in the membrane stabilization or labilization.

### 3.9. Could DHPs Be Involved in Antioxidative Stress?

Before being applied* in vivo*, the optimal dose and optimal time intervals for DHPs application must be known. Namely, while ROS have been traditionally considered as toxic byproducts of aerobic metabolism, we know nowadays that ROS may act as essential signaling molecules, needed for the control of many different physiological processes. Whether the role of ROS will be damaging, protective, or signaling depends on the delicate equilibrium between time- and location-specific ROS production and scavenging. Accordingly, the imbalance of the increased AO potential, so-called antioxidative stress, could be dangerous similar to chronic OS, in particular in case of extended exposure. Inappropriate interventions in the oxidative homeostasis by excessive antioxidants especially in case of chronic exposure to antioxidants might have very negative effects as was published in the ATBC study, showing an increased cancer incidence in smokers treated by excessive beta-carotene [254]. Therefore, overconsumption of any natural or synthetic AO, including DHPs, as dietary supplements or drugs, must be avoided in order to suppress oxidative damage and must not disrupt the well-integrated antioxidant defense network (Poljsak [255] and Poljsak and Milisav [256]). This is especially important when administrating lipid-soluble antioxidants that can be stored in biomembranes, thus not only attenuating or preventing LP but also affecting physiological effects of the natural antioxidants, in particular tocopherol. The interrelationship with the status of endogenous antioxidants/prooxidants should be followed.

DHPs primarily suppress the initiation stages of LP process. They do not entirely stop the LP propagation. Acting synergistically with tocopherol, diludine may prevent pathological excess of ROS production within the lipid moiety of the cellular membranes and LDL. However, due to its low solubility and fast metabolism, its concentration in the cells is low. For that reason, it cannot cause antioxidative stress, even if used for an extended period of time. Thus diludine (diethone) could be only a mild antioxidant; it has potential for restoring the pool of natural antioxidants (as synergist of *α*-tocopherol and polyphenols) in the cells.

Moreover, DHPs CA used for cardioprotection and vasodilatation as commercial drugs in low concentrations are fast metabolized* via* CYP3A4, and for that reason their application does not induce cellular AO stress [53, 160]. However Godfraind and Salomone [257] have postulated no evidence that allows recommending dietary supplementation with antioxidants for the primary or secondary prevention of cardiovascular disease.

So far, there are no reports on antioxidative stress caused by some DHPs, diludine and its analogues. Diludine and its analogues therefore could act as adaptogens supporting hormetic effects of mild oxidative stress. These compounds may act as potential multisided modulators of Yin-Yang cycles of redox and cell functions (the electroplasmic cycle) (Wagner et al. [258]).

## 4. Conclusions

1,4-Dihydropyridines (1,4-DHPs) have broad spectrum of OS modulating activities. DHPs have reducing and lipid peroxidation inhibitor properties, act as reductants in simple chemical systems, and stabilize various biological systems (LDL, mitochondria, microsomes, cells, and tissues) against OS. Examples and peculiarities and mechanisms of antioxidant activity (AOA) and antiradical activity (ARA) as well as stress-protective effect of DHPs including commercial calcium antagonists (CA) were highlighted. These activities depend on various structural parameters related to DHPs (presence and character of substituents), lipophilicity, and depth of the incorporation in the biological membranes. They also depend on the experimental model system for exploring the lipid peroxidation or oxidative stress. Stress-protective effect of some metabolites of CA (nifedipine) is reviewed. Although some DHPs, including CA, have prooxidant properties (on epididymal sperm cells), they can generally be considered as potent antioxidants. Therefore, comparison of the AOA and ARA of different DHPs (mutually and with other AOs) was described in detail. According to the data presented, the DHPs might be considered as bellwether among synthetic compounds targeting OS and as a kind of pharmacological measure for respective field of organic and medicinal chemistry.

## Figures and Tables

**Table 1:** Relative structure-function relationships of calcium antagonists (DHPs, verapamil, and diltiazem) and vitamin E. Effect on oxidative modification of isolated *ex vivo* human low-density lipoprotein using two various oxidation systems (copper (II) ions induced and monocyte induced). Compiled according to data presented by Rojstaczer and Triggle [119].

Compound	Systems of LDL oxidation			
	Copper (II) ions induced system (comparison of three methods)			Monocyte induced cell oxidation system
	Methods			
	Reduction of TBARS level of LDL (relative efficacy)	Degradation of oxidized [^125^I] LDL by J774 macrophages	Relative electrophoretic mobility of LDL on agarose gel	TBARS content of LDL (in %%)
	Relative efficacy (activity rank order (ARO); ARO = I for the most effective); effective inhibitor concentration [IC], in *μ*M			
Amlodipine	+ + (ARO = IV)	+ + (ARO = II–V)	25 *μ*M 50 *μ*M	25 *μ*M (ARO = III–V)
Felodipine	+ + + + + (ARO = I)	+ + + (ARO = I) 25 *μ*M, 97 ± 2%	50 *μ*M	25 *μ*M, 65 ± 9% (ARO = II)
Nifedipine	+ + + (ARO = III)	+ + (ARO = II–V)	10 *μ*M; 50 *μ*M	25 *μ*M, 96 ± 2% (ARO = I)
2-Chloro analog of nifedipine	+ + + + (ARO = II)	—	—	—
4-Nitro analog of nifedipine	—	+ + (ARO = II–V)	—	25 *μ*M (ARO = III–V)
Nitrendipine	+ + (ARO = IV)	—	No effect	—
Verapamil	+ + (ARO = IV)	+ + (ARO = II–V)	—	25 *μ*M (ARO = III–V)
Diltiazem	+ (ARO = V)	—	No effect	—
*α*-Tocopherol (vitamin E)	+ + + + + + (ARO = I)	—	1 *μ*M; 5 *μ*M; 10 *μ*M; 50 *μ*M	—

**Table 2:** Reduction of intracellular ROS in BAECs by CA DHPs. Compiled according to data reported by Cominacini et al. [123].

Compound	Cellular amounts of compounds (in fmol/cell) determining the 50% reduction (IC_50_) in intracellular ROS concentrations
Lacidipine	4.6 ± 0.7
Lercanidipine	9.2 ± 0.7
Amlodipine	15.3 ± 0.8
Nifedipine	16.4 ± 0.7
Nimodipine	17.2 ± 0.9

**Table 3:** Modulation of ROS formation in BAECs by CA (DHPs and verapamil) and vitamin E. Compiled according to data presented by Cominacini et al. [123].

Compound	Method of flow cytometry (reduced 2′,7′*-*dichlorofluorescein diacetate (DCFH-DA) oxidation by ROS)
	Activity rank order (ARO = I for the highest activity; ARO = III for the mindest activity) (Effective [IC]: 1; 5; 10; 50 *μ*M)
Lacidipine	+ + + (ARO = I)
Lercanidipine	+ + (ARO = II)
Amlodipine	No effect
Nifedipine	No effect
Nimodipine	No effect
Verapamil	+ (ARO = III)
*α*-Tocopherol	+ + + (ARO = I)

**Table 4:** Normolipidemic human blood LDL (0.25 mg/mL) *in vitro* oxidation in the presence of 5 *μ*M CuSO_4_ and CA of 3 types (DHPs, verapamil, and diltiazem) and vitamin E. Compiled according to Lupo et al. [129].

Compound	Methods			
	TBARS method (fluorimetry at 515 nm/533 nm, 4 hours preincubation of LDL with compounds and copper (II) ions; 320% TBARS increase in control during 4 h period)		Inhibition of conjugated diene formation (at 234 nm) expressed as prolongation of induction period (in %% of control). *t*_contr_ = 36.8 min.	
	Effective [IC] (in *μ*M): 1 *μ*M; 10 *μ*M; 50 *μ*M	Activity rank order (ARO = I for the highest activity; ARO = VII for the mindest activity)	Effective [IC] (in *μ*M): 1 *μ*M; 5 *μ*M; 10 *μ*M; 50 *μ*M	Activity rank order (ARO = I for the highest activity; ARO = VII for the mindest activity)
Nifedipine	10 *μ*M; 50 *μ*M	ARO = III	5 *μ*M; 10 *μ*M, 150%; 50 *μ*M, 213%	ARO = III
Amlodipine	50 *μ*M	ARO = IV	5 *μ*M; 10 *μ*M, 122%; 50 *μ*M, 138%	ARO = IV–VI
Isradipine	50 *μ*M	ARO = VI	10 *μ*M, 150%; 50 *μ*M, 183%	ARO = IV–VI
Lacidipine	1 *μ*M; 10 *μ*M; 50 *μ*M	ARO = II	5 *μ*M; 10 *μ*M, 192%; 50 *μ*M, 283%	ARO = II
Verapamil	50 *μ*M	ARO = V	10 *μ*M, 150%; 50 *μ*M, 178%	ARO = IV–VI
Diltiazem	No effect	No effect (ARO = VII)	No effect	No effect (ARO = VII)
Vitamin E	1 *μ*M; 10 *μ*M (IC_50_); 50 *μ*M (20% of control)	ARO = I	5 *μ*M; 10 *μ*M, 230%; 50 *μ*M, 370%	ARO = I

**Table 5:** Antiproliferative effect (oxLDL-induced HUVSMCs proliferation) of CA DHPs and simultaneous oxLDL-induced ROS production scavenging. Comparison with N-acetyl-L-cysteine, NAC (intracellular ROS scavenger). Compiled according to data presented by Zou et al., 2012 [130].

DHP compound	Methods			
	Antiproliferative effect against proproliferative effect induced by oxLDL (50 *μ*g/mL) (UV detection of formazan production from tetrazolium salt)		oxLDL-induced ROS production (fluorescent DCF (2′,7′-dichlorofluorescein) production)	
	Effective [IC] in *μ*M and I in %			
Amlodipine	3 *μ*M	I = 18%	3 *μ*M; 10 *μ*M	No effect I = 20%
S(−)-Amlodipine	No effect		No effect	
Lacidipine	10 *μ*M; 30 *μ*M	I = 21% I = 27%	10 *μ*M	I~2/3 of control
N-Acetyl-L-cysteine, NAC	—		5000 *μ*M (5 mM)	I = 28%

## Abbreviations

AD: Alzheimer disease
AO(s): Antioxidant(s)
AOA: Antioxidant activity
ARA: Antiradical activity
CA: Calcium antagonist(s)
CCB: Calcium channel blocker
DHP(s): Dihydropyridine(s)
DNA: Deoxyribonucleic acid
HEH: Hantzsch ester
4-HNE: 4-Hydroxy-2-nonenal
IMAC: Inner membrane anion channel
Lox: Lipoxygenase
LP: Lipid peroxidation
MDA: Malonyl dialdehyde
Mit: Mitochondria
NADH: Reduced nicotinamide adenine dinucleotide
NADPH: Reduced nicotinamide adenine dinucleotide phosphate
NO: Nitrogen oxide
OS: Oxidative stress
PD: Parkinson's disease
RNS: Reactive nitrogen species
ROS: Reactive oxygen species
SOD: Superoxide dismutase
TBARS: Thiobarbituric acid reactive substances
TG: Triglycerides.
